# Oriented Structures for High Safety, Rate Capability, and Energy Density Lithium Metal Batteries

**DOI:** 10.1002/advs.202403797

**Published:** 2024-07-09

**Authors:** Kaiming Wang, Aaron Jue Kang Tieu, Haowen Wu, Fei Shen, Xiaogang Han, Stefan Adams

**Affiliations:** ^1^ Department of Materials Science and Engineering National University of Singapore Singapore 117576 Singapore; ^2^ School of Future Technology Xi'an Jiaotong University Shaanxi 710049 China; ^3^ State Key Laboratory of Electrical Insulation and Power Equipment School of Electrical Engineering Xi'an Jiaotong University Shaanxi 710049 China; ^4^ Xi'an Jiaotong University Suzhou Institute Suzhou Jiangsu 215123 China

**Keywords:** battery safety, energy density, lithium metal batteries, oriented structures, rate capability

## Abstract

Lithium metal batteries (LMBs) have emerged in recent years as highly promising candidates for high‐density energy storage systems. Despite their immense potential, mutual constraints arise when optimizing energy density, rate capability, and operational safety, which greatly hinder the commercialization of LMBs. The utilization of oriented structures in LMBs appears as a promising strategy to address three key performance barriers: 1) low efficiency of active material utilization at high surface loading, 2) easy formation of Li dendrites and damage to interfaces under high‐rate cycling, and 3) low ionic conductivity of solid‐state electrolytes in high safety LMBs. This review aims to holistically introduce the concept of oriented structures, provide criteria for quantifying the degree of orientation, and elucidate their systematic effects on the properties of materials and devices. Furthermore, a detailed categorization of oriented structures is proposed to offer more precise guidance for the design of LMBs. This review also provides a comprehensive summary of preparation techniques for oriented structures and delves into the mechanisms by which these can enhance the energy density, rate capability, and safety of LMBs. Finally, potential applications of oriented structures in LMBs and the crucial challenges that need to be addressed in this field are explored.

## Graphical abstract

Utilizing oriented structures is a promising approach to simultaneously enhance the energy density, rate capability, and safety of Li metal batteries. To guide a rational device optimization, the concept, categories of and preparation methods for oriented structures, and criteria to quantify the degree of orientation are reviewed, and mechanisms for how they affect materials and device performance are discussed.

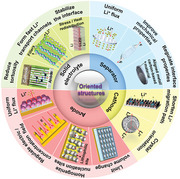

## 1 Introduction

In an effort to reconcile the surging demand for energy supply, while simultaneously conserving the environment, many countries have turned to renewable energy sources such as wind, solar, biomass, geothermal, and hydrogen. However, the contribution from renewable energy, including solar, wind, and hydroelectricity, to global primary energy consumption in 2022 is still relatively moderate at 14.2%, compared to 81.8% from fossil energy sources such as oil, gas, coal, and 4.0% from nuclear energy.^[^ ^1^ ^]^ A major challenge hindering the widespread adoption of renewable energy is the requirement for reliable large‐scale energy storage technology to reduce the security risks posed to power grids by the intermittent, fluctuating, and stochastic nature of renewable energy generation.^[^ ^2^ ^]^ In addition, the increasing adoption of electric vehicles in modern society also prompts high‐energy–density storage devices to urgently eliminate the “range anxiety” problem.

Among the available energy storage, lithium (Li)‐ion batteries (LIBs) are well‐qualified to meet the short‐term expectations of the sustainable energy industry in terms of their high energy conversion rate and relatively long cycle life, but further improvements in energy density remain desirable. Therefore, Li metal batteries (LMBs), where Li metal anodes (LMAs) replace conventional Li‐ion intercalating anodes, are regarded as promising next‐generation high‐energy‐density batteries, due to the tenfold increase in specific capacity (from 372 to 3860 mAh g^−1^), a low negative potential (−3.040 V vs standard hydrogen electrode) and low density (0.534 g cm^−3^) within the LMAs.^[^ ^3^, ^4^, ^5^ ^]^ By optimizing the electrolyte and electrode design, LMBs can achieve energy densities beyond 500 Wh kg^−1^, a 60–100% increase compared to conventional LIBs that typically feature energy densities of 250–300 Wh kg^−1^.^[^ ^4^, ^6^ ^]^ The theoretical energy densities of Li–O_2_, and Li–S batteries are exceptionally high, reaching up to 3505 and 2567 Wh kg^−1^ respectively.^[^ ^5^ ^]^ Hence, from electric vehicles to portable electronic devices, and further to grid storage and aerospace applications, the deployment and utilization of LMBs is expected to yield significant technological and economic benefits.

**Figure 1:**
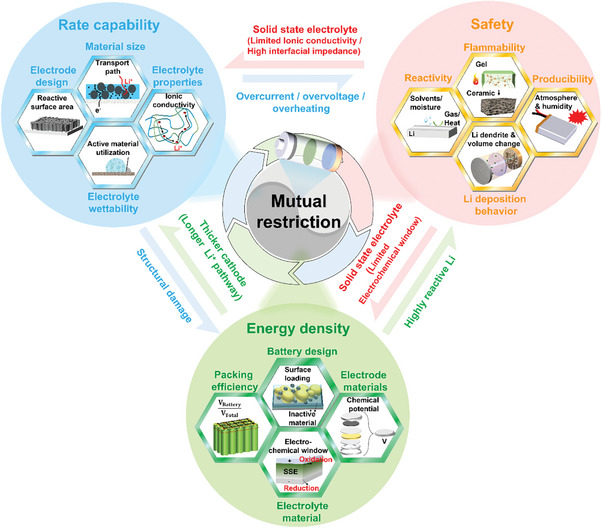
The three main performance indicators for LMBs and their interrelationships (Arrows indicate the effect of improving this performance parameter on the performance parameter in the indicated direction). As an example, the use of solid‐state electrolyte (SSE) improves safety, but the restricted electrochemical window and higher density of SSE tend to limit the practical energy density of the LMBs, as shown by the red arrows.

However, as shown in **Figure** **1**, the energy density indicator of LMBs highlights the mutually constraining relationship with the two other performance indicators. For instance, given that the power density is typically restricted by three limiting factors: 1) ion transport in the electrolyte, 2) charge transfer at the electrode/electrolyte interface, and 3) diffusion inside the electrodes,^[^ ^7^ ^]^ an improvement in the electrolyte wettability of the electrode and the ionic conductivity of the solid‐state electrolyte (SSE) will, on one hand, enhance the rate capability of LMBs. On the other hand, fast cycling rates will tend to cause more pronounced structural damage to the active material and thus make it more difficult to maintain a high energy density over the cycle life. Moreover, the substantial heat generated in this process also poses significant safety concerns, as indicated by the two blue arrows in Figure 1.

Similarly, the green arrows symbolize that increasing the energy density of Li batteries by thicker cathodes with higher loading of active materials and by adopting LMAs will have an adverse effect on the rate capability and safety performance due to longer migration pathways for ionic and electronic charger carriers and exacerbated side reactions. Safety hazards associated with Li metal as anodes may arise by various routes: 1) The high reactivity of Li tends to cause the generation of substantial amounts of solid or gaseous by‐products that may increase the internal resistance, leading to excess heat generation or even fire and explosion. 2) At the relevant rates, Li tends to be deposited in dendritic morphology. The dendrites can penetrate the SSE/separator, causing internal short circuits. 3) The substantial volume fluctuations during cycling inherent to the “host‐less” nature of Li metal anodes may lead to device damage and safety risks.

Therefore, numerous research groups explore various nonflammable SSE including oxides, sulfides, halides, polymers, and their composites as electrolytes to further improve safety performance. However, SSE‐based cells face the challenging task of simultaneously achieving fast ionic conductance and low interfacial impedance with the electrodes while having high electrochemical stability of the SSE layer. These challenges have a direct impact on the cycling rate and the output voltage, ultimately limiting the achievable power and energy density. This relationship is illustrated by the two red arrows in Figure 1. Hence, novel, universally applicable design principles are urgently desired to overcome the mutual constraints in the optimization of battery components and allow to simultaneously enhance energy density, rate capability, and safety performance.

**Figure 2:**
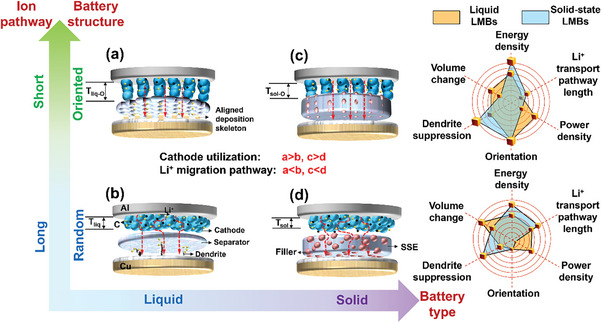
Relationship between degree of orientation and battery performance in liquid or solid‐state LMBs, and battery performance indicators before and after application of oriented structures.

Recently, the adoption of oriented structures in LMBs has gained recognition as an efficient means to enhance energy density without compromising rate capability and safety performance.^[^ ^8^ ^]^ The oriented Li^+^ migration channels sketched in **Figure** **2** enhance the electrolyte wettability of the electrodes, which improves the utilization of the active materials at high surface loading, and thereby increases the energy density. Moreover, cathodes with orientationally aligned active materials and SSE composites with highly oriented fast ion‐conducting particles/fibers/skeleton are capable of remarkably shortening the Li^+^ migration paths in both liquid and solid‐state LMBs. In consequence, this accelerates the kinetics of the electrochemical reaction and thus increases the rate performance. Furthermore, it is generally accepted that power density is also influenced by the structure of the battery components, as Li deposition/stripping will essentially take place at the restricted interface between the electrolyte and Li anode.^[^ ^9^ ^]^ Therefore, 3D structures with various degrees of orientation such as metal skeletons and carbon mats are introduced into LMAs. Figures 2a,c illustrate that a more homogeneous Li^+^ flux and Li nucleation can be realized by introducing oriented structures in a separator, Li anode skeleton, and SSE, which can inhibit the formation of local Li deposition “hotspots” and the growth of Li dendrites. In general, oriented structures can be used in both liquid and solid LMBs, to fulfill a multi‐faceted, comprehensive function, allowing for better balance and adjustment of the relationship between battery energy density, rate capability, and safety performance.

To date, although there have been reviews focusing on enhancing the power and energy density of LMBs through the rational design of oriented structures, there have been limited discussions regarding the 1) systematic evaluation of orientated structures, 2) the classification of orientated structures, and 3) the mechanisms by which the degree of orientation affects the energy, power density as well as the safety performance. In this work, we introduce and establish, for the first time, a detailed evaluation system for oriented structures, comprehensively discussing the different types of oriented structures and their unique functions in all battery components. Then, we categorize the manufacturing techniques for diverse oriented structures and evaluate their potential for commercialization based on factors such as scalability, practicality, cost‐effectiveness, and processing complexity. Subsequent sections delve into the mechanisms by which oriented structures enhance the performance of LMBs. Finally, we discuss challenges arising for the manufacturing technology of oriented structures in LMBs, and present selected new trends to overcome these challenges and open wider fields of future application for oriented structures in LMBs.

## 2 Definition of Oriented Structures in LMBs

Oriented structures in LMBs refer to at least four aspects:

1. The specific arrangement of components inside the battery, such as cathode particles, in a predictable and controlled manner.
2. The specific layout patterns and distribution of additives within the LMBs, such as fillers in SSE, to achieve the maximum performance enhancement with a minimal amount of additive.
3. The optimization of the internal structure of the battery components, such as the orientation alignment of crystalline layers within cathode particles and the pore distribution of the separators.
4. Regular gradual changes in the characteristics of the battery components in certain directions, such as lithiophilicity and conductivity of the deposition skeleton, that influence the Li deposition/stripping behavior.

Overall, it appears necessary to first elaborate an evaluation system for the degree of orientation and a classification index for oriented structures, to guide future research on novel oriented structures.

### 2.1 Evaluation System of Oriented Structures

**Figure 3:**
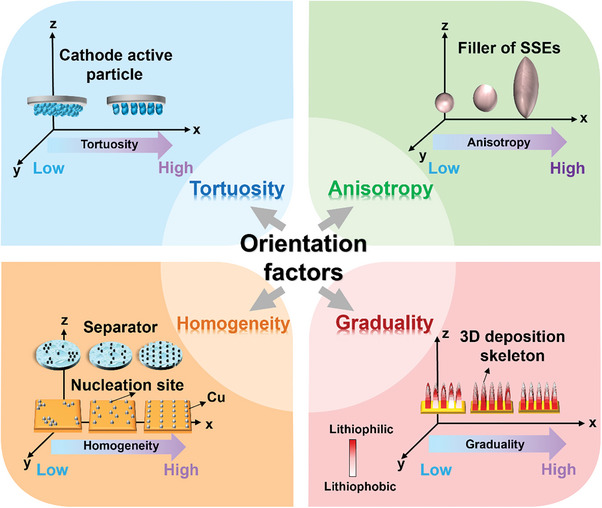
Four indicator categories for characterizing oriented structures.

In the realm of LMBs, achieving high energy density, high power density, and safety simultaneously is difficult and challenging. Nonetheless, the introduction of oriented structures in LMBs offers a promising avenue to address this challenge synergistically from multiple perspectives. Hence, establishing a quantitative evaluation system for oriented structures should enable researchers to more precisely and rationally develop modification methods aligned with the desired optimization goals. As outlined in **Figure** **3**, oriented structures are commonly characterized by an evaluation system consisting of four indicators: tortuosity, anisotropy, homogeneity, and graduality.

#### 2.1.1 Tortuosity

**Figure 4:**
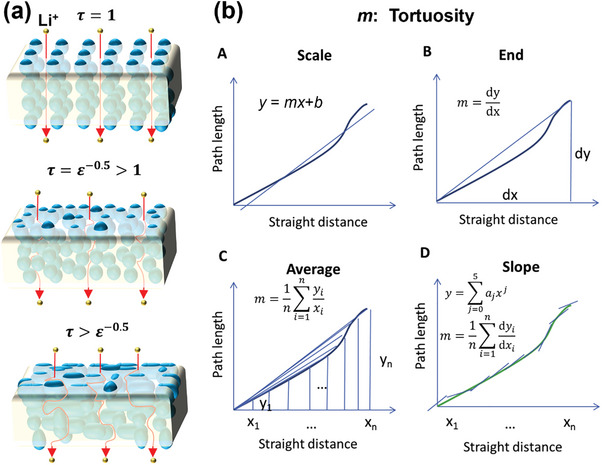
a) Effect of the arrangement of vertically aligned particles, randomly distributed isotropic particles, and randomly distributed anisotropic particles on tortuosity. b) Four different methods to extract the tortuosity from the distance curve. A) Scale: the curve is fitted with the equation *y* = *mx*+*b*, where *x* represents the straight distance, *y* is the path length and the tortuosity is approximated by *m*. B) End: tortuosity is defined as the average path length at the end plane divided by the straight distance. C) Average: tortuosity is defined by the average tortuosity from each plane; tortuosity. D) Slope: tortuosity is extracted by averaging all local slopes. In this case, the local slopes are obtained by first fitting the curve with a polynomial of degree five and then calculating the derivative at each value of *x*. Reproduced with permission.^[^ ^12^ ^]^ Copyright 2014, Elsevier.

Tortuosity (τ) is used to quantify the effect of microstructure on macroscopic effective transport properties.^[^ ^10^, ^11^ ^]^ It is defined as the ratio between the actual path length and the straight distance:^[^ ^12^, ^13^ ^]^

(1) $$\tau =\frac{d_{\mathrm{eff}}}{d}$$

with *d*_eff_ as the effective actual path length from point x to point y, and *d* as their straight (Euclidean) distance. The square of the tortuosity τ is known as the tortuosity factor (κ).^[^ ^12^ ^]^ It should be noted that κ = τ^2^ is sometimes also incorrectly referred to as tortuosity in the literature. A tortuosity of τ = 1 applies, if the conduction pathways consist of straight channels with a uniform cross‐section that are parallel to the transport direction, otherwise τ > 1 (**Figure** **4**a). The connection between the effective diffusivity of ions (*D*_eff_) and the tortuosity of the actual porous structure can be expressed as:^[^ ^7^, ^11^ ^]^

(2) $$\frac{D}{D_{eff}}=\frac{\kappa }{\varepsilon }\;=\frac{\tau^{2}}{\varepsilon }=N_{M}$$

where *ε* is the porosity (generally referred to as the volume of the pores as a fraction of the total volume) and *D* is the diffusion coefficient of ions in the electrochemical system. The ratio *D*/*D*_eff_ defines the so‐called MacMullin number, *N_M_*. These equations indicate that higher porosity and lower tortuosity will promote fast effective ion diffusion.

Notably, there are two distinct approaches on how to calculate tortuosity in actual 3D structures. One method is the “effective diffusion” based on the diffusive transport across one phase divided by the transport flux of the straight path. The tortuosity is then determined according to Equation 2, which requires prior knowledge of the porosity ε. An alternative approach is the “geometric method” based on identifying the length of the tortuous path in the 3D microstructure and dividing it by the length of the straight path according to Equation 1. As shown in Figure 4b, the first way of calculating tortuosity by the “geometric method” is to fit a curve with a single slope, but it can only represent a general trend. Among the remaining three ways belonging to the “geometric method,” named “End,” “Average,” and “Slope,” the closest to the tortuosity calculated by the “effective diffusion” method is “Average” because each of the individual planes is taken into account and the total tortuosity is defined as the average of the tortuosity values from each plane.^[^ ^12^ ^]^

#### 2.1.2 Anisotropy

Anisotropy in this context mainly refers to the size, shape, and arrangement of nanostructured fillers forming a complex porous network, such as in electrodes and SSE.^[^ ^14^ ^]^ An empirical correlation between geometrical variables such as porosity *ε* of a network and its tortuosity *τ* is provided by a generalized Bruggeman‐type relationship:^[^ ^15^, ^16^ ^]^

(3) $$\kappa =\tau^{2}=\gamma \varepsilon^{1-\alpha }$$

where *α* is the Bruggeman exponent, and *γ* is a linear scaling factor. Figure 4a also illustrates that the *α* value of a system composed of a continuous conductive phase mixed with isotropic, uniform size and randomly distributed insulating spherical particles is commonly taken as 1.5 (second graph in Figure 4a), while anisotropic shape and arrangement of the insulating filler particles generally leads to a substantial increase of *α* to values larger than 1.5 (third graph in Figure 4a), thus increasing the tortuosity of the electrodes and SSE.^[^ ^9^, ^14^, ^15^ ^]^ Geometric factors *γ *> 1 will contribute to the increase in tortuosity factor *κ* and MacMullin number *N*_M_. From studying comparable systems over a range of porosities, log(γ) and ‐*α* may be read from linear regression of log (*N*_M_ (*ε*)) versus log (*ε*) plots. Experimental data by Landesfeind et al.^[^ ^9^ ^]^ for a range of porous separators lead to values of *α* ≈ 2.32 and *γ* = 1.39, i.e., ≈2–3 times higher tortuosity factors *κ*, or 2–4 times higher values of *N*_M_ than to be expected from the Bruggeman model. For a range of LFP cathodes, Thorat et al.^[^ ^13^ ^]^ find *α* = 1.53 (close to the Bruggeman model) but *γ* = 1.8, supporting the overall finding that realistic (non‐monodisperse and non‐spherical) systems will have greater tortuosity than predicted by the Bruggeman model. Furthermore, as discussed by Tjaden et al,^[^ ^15^ ^]^ the detailed microstructural characteristics of the porous layer as a consequence of manufacturing technique, composition, particle size, and shape, etc. will control, whether the deviations from the Bruggeman model over an often‐limited range of accessible porosities will affect the tortuosity factor linearly (*γ* > 1) or can be more accurately described by an exponent deviating from *α* = 1.5.

#### 2.1.3 Homogeneity

Homogeneity refers to the arrangement of internal battery components or modified materials in a systematic manner, following a specific rule, direction, and sequence to achieve a particular effect. Typically, microstructures exhibiting high homogeneity display periodic characteristics, enabling the creation of an entire oriented structure through the repetition and alignment of basic units (such as particles, fibers, rods, flakes, or blocks) within the battery.

**Figure 5:**
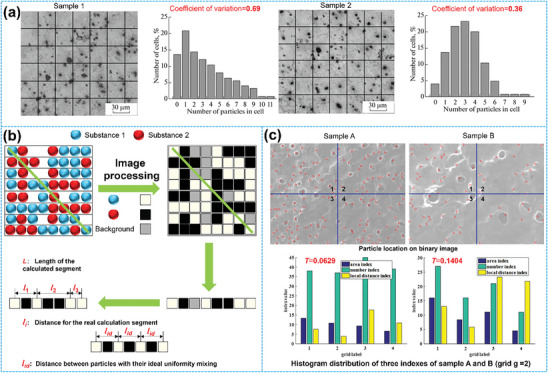
The uniformity assessment criterion of a) the quadrature‐based method. Reproduced with permission.^[^ ^18^ ^]^ Copyright 2022, Springer Nature. b) the distance‐based method. c) a composite of the quadrature‐based and distance‐based methods. Reproduced with permission.^[^ ^17^ ^]^ Copyright 2021, Wiley‐VCH.

Evaluations of material distribution homogeneity are typically conducted by image processing techniques that discretize scanning electron microscope (SEM), transmission electron microscope (TEM), or atomic force microscope (AFM) images by gray‐scale transformation into computer‐recognizable information about the target structure.^[^ ^17^ ^]^ Methods for assessing uniformity based on spatial point models can be categorized into three approaches. The first approach focuses on the quadrature method, which divides the images into a fine grid of cells, called quadrants, determines the phase distribution within each quadrant, and builds a mathematical model to assess the homogeneity of the phase distribution across the quadrants. Kalashnikova et al.^[^ ^18^ ^]^ assessed the degree of dispersion and homogeneity of Ti_2_NiAl particles in an aluminum‐matrix composite by a coefficient of variation *ν* (CV). Composite sample images were segmented and analyzed for the number density distribution of particles in each quadrant (**Figure** **5**a). Thus, the coefficient of variation, ν, is given by Equations ((2), (3), (4)):

(4) $$v\;=\frac{\sigma }{\bar{x}}=\frac{\sqrt{\frac{\sum_{i=1}^{n}{\left(x-\bar{x}\right)}^{2}}{n}}}{\bar{x}}$$

where *σ* is the root mean square deviation, *x* and $\bar{x}$ represent the actual and average number of particles in each quadrant, respectively, *n* is the number of quadrants. Obviously, *υ* = 0 characterizes an ideally uniform particle distribution, and higher values of *v* indicate a tendency toward a nonuniform distribution. For example, it can be seen from Figure 5a that *υ* for Sample 1 is 0.69, which is larger than *υ* = 0.36 for Sample 2, indicating that the Ti_2_NiAl particles in Sample 2 are more evenly distributed. However, the morphological and spatial information about a particular target material is lost in this approach, as only the quantity of that material in the distribution characteristics is considered. In particular, the approach does not allow to draw conclusions on whether the deviations from the average occur randomly, follow a regular pattern, or arise due to an overall systematic gradient across the sample (see graduality in the next section). It should also be noted that the value of this coefficient of variation *ν* depends on the grid resolution chosen, so the resulting value can only specify the homogeneity on a particular pre‐determined length scale.

The second approach is distance‐based, i.e., it mainly relies on the distance between particles to characterize the homogeneity of their distribution. Figure 5b exemplifies this using a schematic diagram of a composite consisting of two types of particles. In an image of such a composite, each pixel point is determined to belong to substance 1, substance 2, or the background substrate, and the homogeneity of mixing for the particles is quantified based on the ratio *R_i_* of the actual distance *l*_i_ between like particles to the expected distance *l*_id_ for an ideally uniform distribution.^[^ ^19^ ^]^ To ensure that the ratio parameter *R_i_* is always between 0 and 1, it is defined by Equation (5) as:

(5) $$R_{i}=\left\{\begin{array}{c} \frac{l_{i}}{l_{id}},\;if\;l_{i}\leq l_{id}\;\\ \frac{l_{i}-L}{l_{id}-L},\;if\;l_{i}\geq l_{id} \end{array}\right.\;$$

where *L* represents the overall length of the linear segments for which the distances are determined. Ultimately, the distribution homogeneity is quantitatively assessed as a degree of mixing (*I* ^`^) defined as:

(6) $$I^{`}=\frac{1}{N}\sum_{i\;=1}^{N}R_{i}$$

where *N* is the number of intervals. Based on this definition perfect homogeneity corresponds to *I* = 1, while *I*≈0 corresponds to an extremely nonuniform distribution. However, this simplistic approach for quantifying homogeneity is more suitable for 1D distributions as it ignores the morphological information on the added substances. Moreover, the functional form, in which the overall segment length *L* influences the ratio parameter *R* may be hard to justify (in general *L* will be much larger than *l*_i_ or *l*_id_, so that deviations from *R* = 1 for *l*_i_ > *l*_id_ will appear much smaller than for *l*_id_ > *l*_i_).

Given the drawbacks of the above methods, the third evaluation method combines the advantages of the first two methods by creating three indicators consisting of composite area, composite number, and local distance within each grid to quantify homogeneity more comprehensively.^[^ ^17^ ^]^ As shown in Figure 5c, the structural information is extracted by binary image processing and morphological transformation. Then, homogeneity is assessed by three metrics *T_l_*, and the homogeneity assessment expression (*T*) is given as the weighted sum of these three metrics:

(7) $$T\;=\sum_{l=1}^{3}\beta_{l}T_{l}\;,\;\left(\;\sum_{l\;=1}^{3}\beta_{l}=\;1\right)$$

The weighting factor *β_l_* highlights the relative importance of a particular index *T_l_*. The default choice of *β_1_* = *β_2_* = *β_3_* = 1/3 turns *T* into the arithmetic mean of the individual *T_l_*:

(8) $$T_{1}=\frac{\sqrt{\frac{\sum_{i=1}^{g\times g}{\left(a_{i}-a\right)}^{2}}{\left(g\times g\right)-1}}}{a}$$

(9) $$T_{2}=\frac{\sqrt{\frac{\sum_{i=1}^{g\times g}{\left(b_{i}-b\right)}^{2}}{\left(g\times g\right)-1}}}{b}$$

(10) $$T_{3}=\frac{1}{g\times g}\sum_{i\;=1}^{g\times g}\frac{d_{i}}{D_{i}}=\frac{1}{g\times g}\sum_{i\;=1}^{g\times g}\frac{\sqrt{{\left({\bar{x}}_{i}-X_{i}\right)}^{2}+{\left({\bar{y}}_{i}-Y_{i}\right)}^{2}}}{D_{i}}$$

Here, *T*_1,_ *T*_2_, and *T*_3_ are CVs of the area ratio, number, and local distance of the composites, respectively. *a_i_* and *a* represent the area ratio of the target material within the *i* ^th^ grid element of the *g* × *g* assessment grid and the average area fraction of that material in each grid, respectively. Similarly, *b_i_* and *b* are the total number of target materials in the *i* ^th^ grid element and the average number of that material across all grid elements, respectively. (*X_i_*, *Y_i_*) indicates the center coordinate of the *i* ^th^ grid element, (${\bar{x}}_{i},\;{\bar{y}}_{i})$ is the average centroid coordinate of the target material within the *i* ^th^ grid. *D*_i_ is the maximum distance between the centroid of each target material in the *i* ^th^ grid and the center of the *i* ^th^ grid. A smaller value of *T* indicates a more homogeneous distribution. Sample A in Figure 5c has a pore distribution homogeneity of *T* = 0.0629, which is clearly smaller than that of sample B with *T* = 0.1404. This indicates that sample A has a more homogeneous pore distribution.

To analyze whether the deviations from homogeneity follow a regular pattern, various methods developed for automated image analysis and denoising have been applied. Typically, they employ assigning a (grayscale) value to each pixel of a 2D image or 3D data volume and conducting a Fourier transformation to determine the respective amplitude of sinusoidal deviations from the uniformity of different wavelengths. They may in principle be viewed as virtual analogs of diffraction experiments that analyze the crystallinity of materials or surfaces.

#### 2.1.4 Graduality

Graduality describes changes in a feature of an oriented structure at the macroscopic scale. One typical example is the design of a skeleton with increasing lithiophilicity in a vertical direction to the Li metal anode surface, which can induce a “bottom–up” deposition pattern to prevent Li^+^ from being deposited on the top of the 3D current collector, thereby suppressing the generation of dendrites and dead Li during cycling and utilizing the space available.^[^ ^20^ ^]^

To quantify the extent of graduality for the simplest case of a linear variation of values of a property *y*_i_ found for a series of *n* quadrants positioned at *x*_i_ along a considered direction X the linear correlation coefficient *R* can be calculated as:^[^ ^21^, ^22^ ^]^

(11) $$R=\frac{\;\sum_{i=1}^{n}x_{i}y_{i}-\left(\sum_{i=1}^{n}x_{i}\right)\left(\sum_{i=1}^{n}\left(y_{i}\right)\right)}{\sqrt{\left[n\;\sum_{i=1}^{n}x_{i}^{2}\;-\;{\left(\sum_{i=1}^{n}x_{i}\right)}^{2}]\times [n\;\sum_{i=1}^{n}y_{i}^{2}-{\left(\sum_{i=1}^{n}y_{i}\right)}^{2}\right]}}$$

#### 2.1.5 Orientational Order in Liquid Crystals and Polycrystals

It may be useful to also consider how the degree of orientational order is commonly characterized in liquid crystalline phases, not only because some of them are ionic crystalline electrolytes, but also because most chain polymers used in energy storage systems can be classified as liquid crystalline materials in the vicinity of their glass transition temperature. The approaches developed for the characterization of liquid crystals may be applied to quantify the extent of orientational order in polycrystalline assemblies of anisotropic crystals.

Liquid crystals or Mesogens generally occur over a limited temperature range between the crystalline state with 3D positional and orientational order (and the isotropic liquid state), especially for molecules that contain both rigid and flexible parts, as the rigid components tend to align mesogen moieties in one direction, while the flexible segments (often alkyl chains) provide mesogens with sufficient mobility to impede crystallization.^[^ ^23^ ^]^ Anisotropic structures such as 1D chains and 2D layers are also frequently observed in solid‐state inorganic materials. Martin et al.,^[^ ^24^ ^]^ e.g., engineered lamellar, cubic, and hexagonal liquid–crystalline structures in metal–halide melts by adding alkylammonium surfactants. Still, so far little is understood about structural organization in the vicinity of the melting point of such inorganic materials.

Varying degrees of order and local mobility lead to different classes of liquid crystalline phases. Among these, *nematic* phases exhibit long‐range orientational order, but only short‐range positional order. In contrast, *smectic* phases possess long‐range orientational order and also some positional order that aligns rod‐shaped molecules into layers. Similarly, molecules with disk‐shaped rigid cores may show a *nematic* phase with preferred orientation but not positional order, and also exhibit *columnar* phases where positional order is realized by stacking the disk‐shaped molecules into roll‐like columns.

In the simplest (and most common) case of uniaxial nematic liquid crystals, the extent of orientational order can be quantified by a scalar orientational order parameter ***S*** that is based on a spatial and temporal average of the second Legendre polynomial P_2_(cosΘ):

(12) $$S=\langle P_{2}\left(\cos\Theta \right)\rangle =\langle \frac{3cos^{2}\left(\Theta \right)-1}{2}\rangle$$

Here Θ is the angle between the axis representing the orientation of an individual liquid‐crystal molecule (which can be the long axis of a linear molecule or the normal to the molecular plane for disc‐shaped molecules) and the local director (i.e., the overall “preferred direction” within a volume element of a liquid crystal sample). Based on this definition the possible values range from *S* = 0 for isotropic samples with a completely random orientation of the molecules to *S* = 1 for perfectly aligned molecules. For typical liquid crystalline samples, values of *S* are commonly found to be in the range of 0.3–0.8 and decrease with increasing temperature, eventually reaching 0 at the phase transition temperature of the isotropic liquid phase. Depending on the nature of the molecules, *S* can be determined experimentally by measuring order‐dependent properties of the materials such as diamagnetism, birefringence, Raman scattering, NMR, and EPR.^[^ ^25^ ^]^

In the characterization of polycrystalline assemblies, the extent of orientational order in the distribution of crystallographic orientations of the crystallites is commonly termed *texture*.^[^ ^26^, ^27^ ^]^ The quantification of texture in polycrystalline samples is typically conducted by X‐ray or Neutron diffraction using texture goniometers, or by electron backscatter diffraction (EBSD) method in scanning electron microscopes. A full 3D representation of crystallographic texture is given by the orientation distribution function (odf), defined as the volume fraction of crystallites with a certain orientation ***g***:

(13) $$odf=\frac{1}{V}\;\frac{dV\left(g\right)}{dg}$$

***g*** itself is characterized by three Euler angles describing the transition from the sample's axis system into the crystallographic axis system of each individual crystallite. Texture is commonly visualized using *pole figures* as a stereographic projection of the frequency distribution of crystallite orientations relative to a specified crystallographic axis (or pole) of interest.

As a simpler scalar measure of the degree of orientational ordering of crystallite arrangements, the March–Dollase pole density profile is given by the *preferred orientation factor* (*R*).^[^ ^28^ ^]^ The relative frequency *O* of crystal orientations with a given polar angle *ρ* between a specified preferred orientation direction, $\vec{p}$, of a crystallite and the specimen direction, $\vec{s}\;$ is expected to follow Equation (14):

(14) $$O\;\left(R,\rho \right)={\left(R^{2}cos^{2}\left(\rho \right)+\;sin^{2}\left(\frac{\rho }{R}\right)\right)}^{-\frac{3}{2}}$$

This formula assumes that the crystallite shapes can be approximated as ellipsoids with rotational symmetry around $\vec{p}$. In the original derivation of the orientational distribution of such ellipsoids, *R* refers to the ratio of the ellipsoid diameter along and perpendicular to $\vec{p}$. *R* = 1 corresponds to the random arrangement of spherically shaped crystallites, while values of 0 < *R *< 1 correspond to plate‐shaped crystallites that preferentially orient parallel to the sample surface (i.e., The normal $\vec{p}$ to the crystallite surface is perpendicular to the sample surface), while *R* > 1 indicates needle‐shaped crystals that preferentially orient with the needle axis $\vec{p}\;$ parallel to the sample surface. Despite its approximate character for real crystallite shapes, it is commonly applied for Rietveld analyses of polycrystalline ceramics. For strongly textured samples induced by mechanical processing (e.g., the rolling and recrystallization textures of fcc metals), more elaborate descriptions by means of spherical harmonics are required to obtain accurate results.^[^ ^26^ ^]^

### 2.2 Classification of Oriented Structures

The classification of oriented structures holds significant value in battery design, providing essential guidance for advancing battery technology and expanding its applications. Selecting the most suitable modification method to accurately design the oriented structure enables improvements in energy density, rate capability, and safety performance of the LMBs in the future. To date, no classification systems and methodologies have been proposed to explicitly and comprehensively categorize the oriented structures in LMBs. Two methods are introduced in this paper to classify oriented structures based on the battery components concerned and the spatial dimensionality, respectively.

#### 2.2.1 Classification According to Battery Components

**Figure 6:**
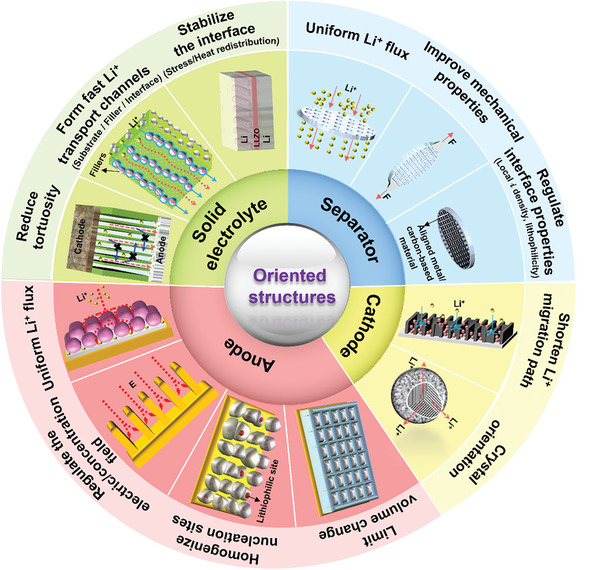
Classification of oriented structures in LMBs according to the affected battery components and their respective functions.

Oriented structures occur universally in all battery components and each of them has the potential to significantly affect the performance of LMBs (as shown in **Figure** **6**). They can be employed across various scales, ranging from nano‐ to macro‐scale, to tailor the safety and energy/power density of the battery. Furthermore, the application of oriented structures to specific battery components yields synergistic effects aimed at multiple optimization objectives. For example, the oriented arrangement of fillers in SSE not only enhances its ionic conductivity but also homogenizes the Li^+^ flux and thereby helps to create a dense deposition morphology. The application of oriented structures in different battery components may result in comparable overall effects at different costs. Therefore, selecting the most suitable optimization target and method should also consider factors such as process complexity and cost.

**Table** **1** provides a detailed comparison of applications of oriented structures in LMBs according to the battery components, including the cathode,^[^ ^29^, ^30^, ^31^, ^32^, ^33^, ^34^, ^35^, ^36^, ^37^, ^38^, ^39^, ^40^, ^41^, ^42^, ^43^, ^44^, ^45^, ^46^ ^]^ anode,^[^ ^47^, ^48^, ^49^, ^50^, ^51^, ^52^, ^53^, ^54^, ^55^, ^56^ ^]^ separator,^[^ ^57^, ^58^, ^59^, ^60^, ^61^, ^62^ ^]^ and SSE.^[^ ^63^, ^64^, ^65^, ^66^, ^67^, ^68^, ^69^, ^70^, ^71^, ^72^, ^73^, ^74^, ^75^, ^76^ ^]^ As a general rule, balancing the energy density, power density, and safety of LMBs through oriented structures can be achieved through the following routes: 1) by modifying various kinetic and thermodynamic behaviors of the Li^+^, including de‐inserting, inserting, diffusion, migration, nucleation, deposition and stripping behavior; 2) by modifying the current density, heat transfer, stress distribution and concentration polarization; and (3) by modifying the mechanical properties of separators or SSE.

**Table 1:** Detailed comparison of oriented structures in LMBs classified by the affected battery components.

	Oriented target	Interaction mechanism	Performance		Ref.
Cathode	Crystalline facets	Shorten Li^+^ migration paths. Suppress crack generation and particle disintegration caused by stress/volume changes. Avoids internal ion dissolution (Ni/Co/Mn) by maintaining structural integrity.	Capacity retention	99.6% after 100 cycles	[77]
				1C, 83% after 1000 cycles	[31]
				0.1C, 91.2% after 100 cycles	[78]
				1C, 95.5% after 300 cycles	[34]
				0.5C, 79.1% after 200 cycles	[35]
				1C, 90.6% after 100 cycles	[36]
	Cathode particles	Shorten Li^+^ diffusion paths. Improved utilization of active material. Alleviate irreversible phase transition.	Mass loading	70–100 mg cm^−2^	[79]
				73.5 mg cm^−2^	[39]
				25–60 mg cm^−2^	[40]
				128 mg cm^−2^	[80]
				99.56 mg cm^−2^	[41]
				72 mg cm^−2^	[43]
Anode	Artificial surface layer (Particle/fiber/rod)	Modulate electric/ion concentration field. Regulate Li nucleation sites and deposition morphology. Accommodate electrode volume change. Reduce local current density. Change the growth direction of deposited Li.	Coulombic efficiency / Cycling time	≈98 % after 400 cycles, 1 mA cm^−2^	[50]
				≈98 % after 1200 cycles,0.5 mA cm^−2^	[54]
	Deposition host			99.08 % after 150 cycles, 5 mA cm^−2^	[49]
				≈150 h at 3 mA cm^−2^	[51]
	Li metal structure			≈99 % after 150 cycles,0.5 mA cm^−2^	[81]
				≈99.8 % after 500 cycles, 1C	[82]
Separator	Artificial surface layer (Particle/fiber/rod)	Uniform Li^+^ flux. Improve mechanical properties. Regulate nucleation sites and depositional morphology.	Coulombic efficiency / Cycling time	98.5 % after 700 cycles, 1 mA cm^−2^	[61]
				98.8 % after 350 cycles, 1 mA cm^−2^	[60]
	Separator structure			≈30 % extension of cell life	[58]
SSE	Skeleton	Provide fast migration channels. Adjust interface stress/heat/current distribution. Regulate nucleation sites and depositional morphology.	Ionic conductivity / Cycling time	2.3 × 10^−4^ S cm^−1^ at 30 °C (Nanoporous polyimide film/PEO)	[64]
				1.8 × 10^−4^ S cm^−1^ at RT (Aligned garnet membrane obtained by wood‐template/PEO)	[69]
				1.16 × 10^−4^ S cm^−1^ at 30 °C (Oriented framework of Al‐doped LLZO nanofiber/PEGDA)	[83]
				5.82 × 10^−3^ S cm^−1^ at RT (Surface‐modified anodized aluminum oxide/PEO)	[70]
	Fillers			0.52 × 10^−4^ cm^−1^ at RT (Vertically aligned and connected LATP nanoparticles/PEO)	[74]
				1.16 × 10^−4^ S cm^−1^ at 30 °C (Aligned LLZO nanofiber films obtained through electrospinning method/PVDF)	[65]
				1.17 ×10^−3^ S cm^–1^ at 30 °C (Vertically aligned LAGP obtained by ice‐templated method/SN)	[73]
				4.7 × 10^−4^ S cm^−2^ at RT (Aligned BN platelet obtained by 3D print/PEO)	[84]
	SSE structure			Li||Li cell stable cycling over 500 h under 0.5 mA cm^–2^	[66]

#### 2.2.2 Classification According to the Structural Spatial Dimension

Classifying oriented structures according to their dimensionality from 0D to 3D is another effective way to distinguish them. In general, 0D structures are commonly referred to as “nanoparticles” or “nanocrystals.” They exist as points in 3D space, lacking a distinct orientation. Therefore, 0D structures are not classified as oriented structures in this context. 1D‐oriented structures refer to wire‐like materials, where atoms are regularly arranged in one direction, while the regular atomic arrangement remains restricted in the other two directions. 2D‐oriented structures refer to ribbon/sheet‐like materials, that extend in two spatial dimensions, while they remain restricted in one dimension. Lastly, 3D‐oriented structures refer to 3D materials that span all three spatial dimensions. As shown in **Table** **2**, the oriented structures are classified in detail combining the above principles of dimensionality, affected components, and the special functions performed by the oriented structures in LMBs. The majority of the oriented structures employed in LMBs are categorized as 2D and 3D structures.

**Table 2:** Classification of orientation structures by dimensionality.

Dimensionality	Characterization	Oriented Target	Interaction mechanism	Ref.
1D	High aspect ratio. Highly ordered linear alignment. Typical structures: nanowires, nanorods.	Properties of Li deposition hosts.	Form conductivity/lithiophilicity gradients. Guide a bottom–up Li deposition pattern/Suppress dendrite growth. Ensure ultralong‐term stable Li stripping/plating.	[20, 85, 86, 87]
2D	Large surface area‐to‐volume ratio. Provide more active sites. Typical structures: nanosheets, graphene, thin films.	Artificial interphase layer (nanometer to sub‐nanometer thickness)	Influence Li^+^ flux and nucleation sites. Increase the contact area between the electrode and the electrolyte. Improve the uniformity and stability of electrochemical reactions.	[48, 50, 54, 61]
3D	Complex internal network structure with porosity. High specific surface area. Provide multidimensional Li+ transport channels. Typical structures: porous frameworks, nanonets, 3D graphene foams.	1) Active cathode particles	Increase utilization of active materials. Improve specific capacity stability at high surface loadings. Mitigate volume expansion and microcrack formation.	[32, 33, 37, 40, 44, 45, 80, 88]
		2) Facet orientation of cathode particles	Establish fast Li^+^ migration channels. Alleviate the volume‐change‐induced inter‐grain stress. Improve Li^+^ diffusion coefficient.	[29, 30, 31, 34, 35, 36, 38, 89]
		3) Solid state electrolyte	Provide fast Li^+^ migration channels. Modify local current density, stress, and heat distribution. Influencing Li nucleation sites and deposition morphology.	[63, 64, 65, 72, 73, 74, 75, 76, 90, 91, 92]
		4) Separator	Improve the mechanical properties. Influencing Li nucleation sites and deposition morphology.	[58, 59, 60, 61, 93]
		5) Artificial interphase layer (Micron thickness)	Influencing Li nucleation sites and deposition morphology.	[53, 56]
		6) Li deposition host	Accommodate volume changes during Li deposition/stripping. Influence on Li deposition patterns (Direction /Morphology).	[49, 55, 81, 94, 95]

##### 1D Oriented Structures

**Figure 7:**
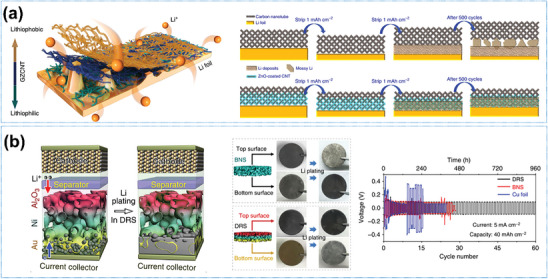
1D oriented structure with some property of regular gradient in one dimension. a) Schematic diagram of the structure consisting of a bottom lithiophilic ZnO/carbon nanotube sublayer and a top lithiophobic carbon nanotube sublayer (left‐hand side) and Li stripping/plating mechanism of Li foils coated with this interfacial layer (right‐hand side). Reproduced under terms of the CC‐BY license.^[^ ^87^ ^]^ Copyright 2018, Springer Nature. b) Bottom–up Li deposition in a deposition‐regulating scaffold (DRS) anode. After plating ≈5 mA h cm^−2^ Li, the morphology changes of the deposition‐regulating scaffold and bare nickel scaffold. Right‐hand side: Comparison of the voltage profiles at an ultrahigh capacity of 40 mA h cm^−2^ at 5 mA cm^−2^. Reproduced under terms of the CC‐BY license.^[^ ^85^ ^]^ Copyright 2019, Springer Nature.

1D‐oriented structures are conventionally defined as structures with only one dimension beyond the nanoscale, but their scope can also be extended to comprise phases, for which specific properties, such as lithiophilicity and electronic conductivity, vary regularly in one dimension. For instance, Zhang et al.^[^ ^87^ ^]^ proposed a lithiophilic–lithiophobic oriented gradient layer with a bottom lithiophilic ZnO/CNT sublayer and a top lithiophobic carbon nanotube sublayer (**Figure** **7**a). This oriented variation of the lithiophilicity properties in a 1D direction achieves a “bottom–up” Li deposition pattern and thus effectively inhibits dendrite growth, eventually ensuring “ultralong‐term stable Li stripping/plating.” Similarly, Pu et al.^[^ ^85^ ^]^ modified the electrical conductivity and lithiophilicity by coating the top of a nickel scaffold (facing the cathode) with Al_2_O_3_ and coating the bottom (facing the current collector) with Au to induce both a conductivity and a lithiophilicity gradient (Figure 7b), thus guiding Li preferentially to deposit densely at the bottom of the deposition‐regulating scaffold (DRS). Photographs of the DRS and bare nickel scaffold before and after plating of ≈5 mA h cm^−2^ of Li confirm the preferential Li deposition behavior due to the lower nucleation barrier for Li at the Au‐coated bottom of the skeleton. Therefore, when the host electrode was pre‐deposited with 40 mA h cm^−2^ Li and assembled into a symmetrical cell, it could still cycle for 60 cycles stably at a current density of 5 mA cm^−2^.

##### 2D Oriented Structures

**Figure 8:**
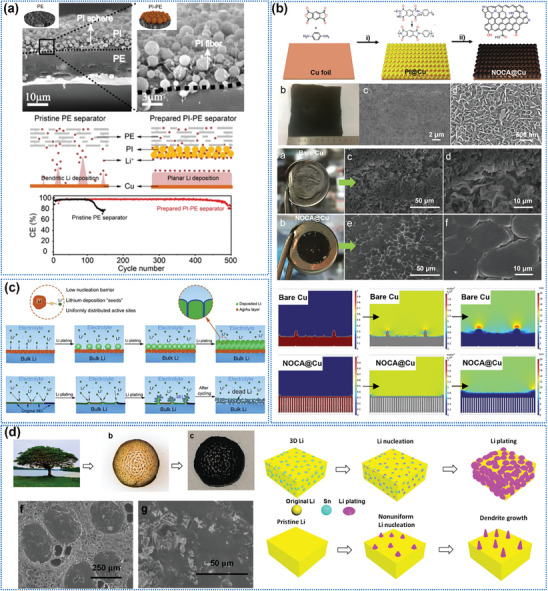
2D oriented structures and their functions. a) Composite 2D‐oriented PI particles layer on the separator surface to modulate Li^+^ deposition behavior. Reproduced with permission.^[^ ^61^ ^]^ Copyright 2021, American Chemical Society. b) Construct nitrogen–oxygen‐doped oriented vertical carbon nanosheet arrays as hosts on Cu foil to effectively suppress Li dendrite growth. Reproduced with permission.^[^ ^54^ ^]^ Copyright 2021, Wiley‐VCH. c) Growing an oriented distribution of Ag particle layers in situ on the surface of the Li metal anode. Reproduced with permission.^[^ ^48^ ^]^ Copyright 2020, Elsevier. d) Image of the tree, 3D wood, 3D carbon, and 3D carbon infused with Li/Sn. And the plating process of 3D Li and pristine Li. Reproduced with permission.^[^ ^96^ ^]^ Copyright 2021, American Chemical Society.

Typical examples of 2D‐oriented structures are interfacial layers formed by organic polymers, carbon‐based materials metals, etc. that are used to regulate the Li^+^ deposition and stripping behavior at the Li metal anode and electrolyte/separator interface. Yin et al.^[^ ^61^ ^]^ compounded orientationally distributed polyimide (PI) particles on the surface of Polyethylene (PE) separators by high‐voltage electrospinning (**Figure** **8**a). The PI particle layer acts through its uniformly distributed pores and Li^+^ adsorption function to create homogeneous nucleation sites, causing the Li deposits to grow in planar islands rather than in dendritic morphology. As a result, the life spans of Cu||PI‐PE||Li half‐cells have been extended to become seven times longer than those of Cu||PE||Li half‐cells at a current density of 1 mA cm^−2^ with a deposition capacity of 0.5 mA h cm^−2^.

Alternatively, 2D‐oriented carbon‐based arrays, such as vertical carbon nanosheets,^[^ ^54^ ^]^ are also found to effectively suppress the growth of Li dendrites. As shown in Figure 8b, both optical and SEM images show a flatter island Li deposition morphology after introducing a vertical carbon nanosheet on a Cu foil current collector. Finite element simulations reveal that this 2D‐oriented structure guides the Li plating into the confined space constructed by the nanoarray and homogenizes the ion concentration and electric field distribution around the electrode.

Compounding 2D‐oriented lithophilic metal sites can also be applied to suppress Li dendrite formation. Liu et al.^[^ ^50^ ^]^ exhibited an in‐situ growth of a layer of homogenously distributed Ag particles oriented on a Cu surface based on the spontaneous substitution reaction between Cu atoms and Ag^+^ ions. The strong lithophilicity of uniformly distributed Ag particles contributes to homogenous Li deposition/stripping, resulting in a high CE of ≈98% even after 400 cycles at 1 mA cm^−2^. Guo et al.^[^ ^48^ ^]^ achieved a dendrite‐free deposition by growing an oriented distribution of Ag particle layers in situ on the surface of the Li metal anode through a substitution reaction between Li atoms and Ag^+^ (Figure 8c). Afterward, Adams’ group^[^ ^96^ ^]^ formed a 3D composite Li metal anode by infusing Li into vertically oriented holes in Sn‐modified carbonized wood (Figure 8d). Sn as the lithiophilic element lowers the formation energy and increases the diffusion coefficient to form a dense and uniform Li deposition morphology.

##### 3D Oriented Structure

**Figure 9:**
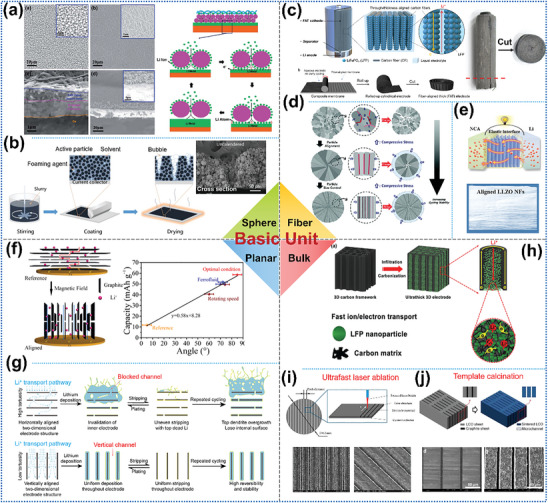
Classification of 3D‐oriented structures by the morphology of the basic unit. a) SEM images of the oriented polyimide particles and the mechanism of uniforming Li^+^ flux. Reproduced with permission.^[^ ^53^ ^]^ Copyright 2020, Royal Society of Chemistry. b) Vertical aggregation of bubbles in cathode slurry by thermal decomposition of NH_4_HCO_3_ to form an oriented arrangement of active particles. Reproduced with permission.^[^ ^97^ ^]^ Copyright 2021, Wiley‐VCH. c) The fabrication process of the oriented carbon fiber as the cathode skeleton. Reproduced with permission.^[^ ^80^ ^]^ Copyright 2020, American Chemical Society. d) Cathode particles composed of oriented rod‐like primary particles. Reproduced with permission.^[^ ^78^ ^]^ Copyright 2020, Wiley‐VCH. e) Oriented ceramic LLZO nanofiber adopted as SSE skeleton to generate fast Li^+^ transport path. Reproduced with permission.^[^ ^65^ ^]^ Copyright 2019, Elsevier. f) Oriented graphite flakes as cathode skeleton and the quantitative relationship between the specific capacity and the angle of alignment. Reproduced with permission.^[^ ^95^ ^]^ Copyright 2019, American Chemical Society. g) Graphene oxide (GO) sheets are employed as basic units to form a 3D‐oriented structure to lower the electrode tortuosity. Reproduced with permission.^[^ ^49^ ^]^ Copyright 2020, Elsevier. h) Carbonized natural wood as a skeleton for the cathode. Reproduced with permission.^[^ ^94^ ^]^ Copyright 2017, Wiley‐VCH. i) Ultrafast laser ablation (Reproduced under terms of the CC‐BY license.^[^ ^98^ ^]^ Copyright 2019, Multidisciplinary Digital Publishing Institute.) and j) laminate template co‐sintering methods to form oriented channels in electrodes. Reproduced with permission.^[^ ^100^ ^]^ Copyright 2020, Elsevier.

3D‐oriented structures are the most widely used and versatile oriented structures in LMBs. As shown in **Figure** **9**, the basic units that compose the 3D‐oriented structure can be divided into 1) sphere, 2) fiber/rod/stick, 3) planar/flake, and 4) bulk/block.

For the spherical basic unit, Liu et al.^[^ ^56^ ^]^ oriented SiO_2_@PMMA core–shell nanospheres to form a 3D interfacial layer for uniform Li^+^ flow, resulting in a flat and even Li deposition morphology. The same effect can be achieved by electrostatic spinning of uniformly oriented layers of polyimide particles compounded with Cu collectors. The uniformly distributed pores of the particle layer and the adsorption of Li^+^ by the PI particles act synergistically to make Li deposition more homogeneous (Figure 9a).^[^ ^53^ ^]^ In addition, the oriented arrangement of active particles in the electrodes can significantly reduce the ion/electron migration tortuosity, thus maintaining a high specific capacity of the active substance even at increased electrode thickness and high cycling rate. For instance, Xiong et al.^[^ ^97^ ^]^ added NH_4_HCO_3_, which thermally decomposed (at 36 °C) to produce vertically aggregating bubbles within the cathode slurry during the drying process, resulting in the formation of microchannels with controlled orientation inside the final cathode (Figure 9b). The rate capability of this modified cathode is increased by approximately seven times at 5C due to the reduced tortuosity of Li^+^ migration paths by the oriented holes. This turns out to be crucial for overcoming the bottleneck of limited ion transport into and out of thick electrodes in high‐energy‐density LMBs.

Shortening the ion/electron migration paths by reducing the tortuosity can also be achieved by arranging fiber‐, rod‐ or stick‐shaped basic units into 3D oriented structures. In particular, carbon fibers show excellent overall performance: in addition to their high electronic conductivity, they are also corrosion‐, abrasion‐, high temperature‐resistant, lightweight, and have high mechanical strength, making them ideal for use as electrode substrates. Figure 9c demonstrates a composite‐oriented carbon fiber substrate cathode with low tortuosity for fast electrolyte infiltration and electron/ion transport.^[^ ^80^ ^]^ After embedding LiFePO_4_ nanoparticles in between carbon fibers, the thickness and active mass loading of this cathode can be increased to ≈1 mm and 128 mg cm^−2^, respectively, while retaining a high specific capacity of 155 mA h g^−1^ at a current density of 0.5 mA cm^−2^. Moreover, modulation of the internal structure of the active material particles at the nanoscale is also a fruitful approach to reducing Li^+^ migration tortuosity. Ryu et al.^[^ ^78^ ^]^ have shown that boron doping in Li(Ni_x_Co_y_B_1−x−y_)O_2_ (NCB) enables the formation of cathode particles composed of an oriented arrangement of rod‐like primary particles with relatively uniform sizes and shapes (Figure 9d). The radial channel formed by this oriented structure facilitates Li^+^ migration and effectively reduces the anisotropic strain to inhibit microcrack formation, improving the cycling stability of the cathode.

Furthermore, the application of 3D substrates consisting of oriented fibers in SSE to obtain fast and continuous ion transport channels is a prominent optimization strategy. For example, Zhao et al.^[^ ^65^ ^]^ prepared an elastic SSE with a high ionic conductivity of 1.16 × 10^−4^ S cm^−1^ at 30 °C by filling the ion‐conducting polymer polyvinylidene fluoride (PVDF) into oriented Li_6.4_La_3_Zr_2_Al_0.2_O_12_ (LLZO) nanofiber membranes (Figure 9e).

Orienting planar or flake‐shaped basic units to form 3D structures also yields significant effects on Li^+^ migration behavior and deposition behavior. As an example, applying a magnetic field modifies the degree of alignment of graphite flakes inside electrodes, thereby affecting their rate performance.^[^ ^95^ ^]^ There is an inverse relationship between the orientation degree and the length of the Li^+^ transport path: A high degree of vertical orientation favors the rapid diffusion of Li^+^ into the electrode (Figure 9f). Thus, the discharge‐specific capacity turns out to be proportional to the angle of alignment. In addition, there is a close correlation between the host tortuosity of Li metal anodes and cycling reversibility. Cui et al.^[^ ^49^ ^]^ investigated three types of deposition hosts: vertically aligned, horizontally aligned, and randomly oriented reduced graphene oxide (rGO) hosts with tortuosity values of 1.25, 4.46, and 1.76, respectively, and concluded that a high degree of host tortuosity leads to an uneven surface deposition of Li and thus reduced cycling performance, while low tortuosity mitigates locally enhanced concentration gradients and leads to a uniform Li distribution across the anode (Figure 9g).

Natural or artificially modified bulk basic units with oriented structures can be used directly as 3D battery components for the purpose of reducing the tortuosity and ion/electron migration paths within the LMBs. As shown in Figure 9h, inspired by the fact that natural wood materials contain channels oriented in the direction of tree growth, Hu et al.^[^ ^94^ ^]^ prepared a highly conductive and lightweight template by direct carbonization of natural wood and compositing it with LiFePO_4_ to form an ultrathick 3D‐oriented cathode. This cathode with a mass loading of 60 mg cm^−2^ active material delivers capacity retention of 76% and high CE of over 99.6% after 140 cycles at 2 mA cm^−2^, much better than that of a conventional thick cathode with only 15% capacity retention after 100 cycles. In addition, oriented channels can also be formed directly in electrodes via ultrafast laser ablation (Figure 9i)^[^ ^98^ ^]^ and laminate template co‐sintering methods to enhance electrolyte wettability and active material utilization (Figure 9j).^[^ ^99^ ^]^ However, laser and high‐temperature calcination have the potential to destroy the active material, so they should be employed with consideration of the actual situation.

### 2.3 Challenges and Limitations in Applying Oriented Structures in LMBs

Oriented structures denote an organized and systematic arrangement of functional fillers and electrode active materials within a battery to create a distinct structure or the direct modification of the intrinsic structure of the electrode, SSE, and separator. Such structures can significantly affect the performance of the LMBs, including the energy density, power density, cycle life, and charge/discharge rate. However, the application of oriented structures in LMBs still poses several challenges and limitations, including:

1. Economical: The cost of oriented structure preparation technologies in large‐scale production should be further reduced by optimizing production processes and developing more affordable alternative materials to increase productivity and efficiency. For example, 3D printing has advantages in terms of freedom in cell structure design, but production efficiency and cost are still not comparable to traditional cell preparation methods such as blade coating of electrodes.^[^ ^101^ ^]^
2. Stability: The ability of oriented structures in LMBs to resist corrosion and abrasion as well as to maintain good stability over long periods of cycling can be a major challenge. In addition, for thick electrodes with oriented structures, mechanical instability of the electrode still remains an issue that needs to be addressed.^[^ ^102^ ^]^
3. Reproducibility: Achieving stable and reproducible oriented structures in LMBs production is challenging, as minor changes in the manufacturing process or experimental conditions can have a significant impact on the degree of orientation of the structure. In the case of separators prepared by the electrospinning method, a slight increment in humidity can lead to the formation of bead‐like structures, which will affect battery performance.^[^ ^103^ ^]^ Therefore, it is essential to ensure the consistency and stability of various processes and environmental parameters in the production preparation and assembly process.
4. Compatibility with mass production: Although technologies such as laser manufacturing and 3D printing have been extensively investigated and applied in the preparation of small battery components, the so far limited knowledge of their practical applicability in the production process chain has prevented their use in state‐of‐the‐art industrial battery production. For example, poor mechanical integrity of electrodes and impurity residues on electrode surfaces after laser treatment remain major challenges for the integration of laser structuring technology into industrial cell manufacturing.^[^ ^104^ ^]^

## 3 Techniques for Fabricating Oriented Structures

Various methods exist to achieve the balance between the energy density, rate capability, and safety of LMBs by adjusting the electrode orientation structure and the arrangement of active materials/functional fillers. Given that each method possesses its own set of advantages and limitations, the selection of the most suitable fabrication technology should be tailored to meet specific requirements in the context of the desired application. Hence, this section elaborates on the effects achieved by each method in LMBs. It should be noted that only techniques that retain the template and do not involve its removal through processes like sintering or sublimation to control the structure of LMBs’ components are classified as template methods in this study.

### 3.1 3D Printing for Oriented Structures in LMBs

**Figure 10:**
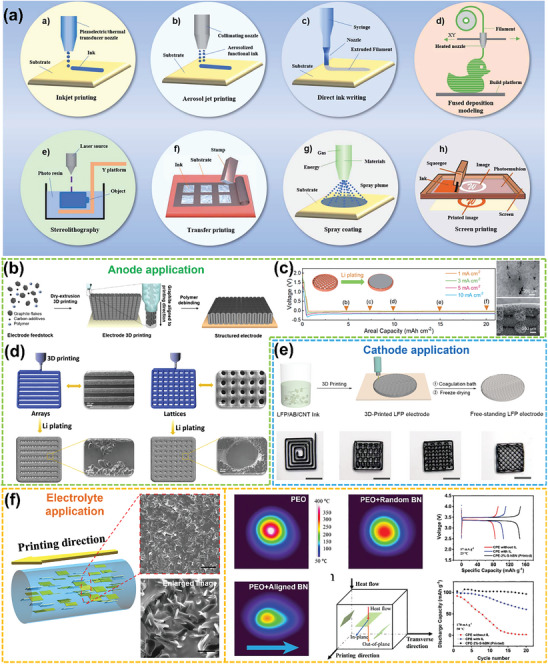
3D Printing techniques for oriented structure in LMBs. a) Overview of printing techniques. (Reproduced with permission.^[^ ^105^ ^]^ Copyright 2022, Royal Society of Chemistry) b) Extrusion‐based 3D Printing technique for graphite anode fabrication. (Reproduced with permission.^[^ ^107^ ^]^ Copyright 2022, American Chemical Society) c) Extrusion‐based DIW 3D printing technique for preparing oriented structured Cu frameworks as skeletons of LMAs. (Reproduced with permission.^[^ ^108^ ^]^ Copyright 2020, Royal Society of Chemistry) d) 3D ink printing of MXene anodes. (Reproduced with permission.^[^ ^47^ ^]^ Copyright 2020, Elsevier) e) 3D printing of ultrathick 3D‐patterned LiFePO_4_ cathode. (Reproduced with permission.^[^ ^109^ ^]^ Copyright 2018, American Chemical Society) f) Fabrication of composite PEO polymers with oriented thermal conductivity BN to uniformly distribute interfacial heat and thereby inhibit dendrite formation by 3D DIW printing technology. (Reproduced with permission.^[^ ^84^ ^]^ Copyright 2020, Wiley‐VCH).

Printing techniques have attracted significant attention due to their high manufacturing precision and simplicity in designing intricate structures, resulting in a great potential for developing oriented structures within LMBs. A typical 3D printing process involves creating a virtual 3D model by the software, slicing it into 2D cross‐sections, and printing the slices layer by layer using a 3D printer.^[^ ^101^ ^]^ As shown in **Figure** **10**a, the most widely applied among the eight printing techniques for preparing oriented electrodes and SSEs are direct ink writing (DIW)/fused deposition modeling and stereolithography, belonging to extrusion‐based and photocuring‐based 3D printing techniques, respectively.^[^ ^105^, ^106^ ^]^

A shear‐assisted particle extrusion technique combined with a rapid sintering process has been used to fabricate thick graphite anodes using graphite flakes oriented along the printing direction (Figure 10b), which reduces electrode tortuosity and ion/electron migration distance to accelerate kinetic processes.^[^ ^107^ ^]^ Beyond the preparation of anodes of graphite‐based LIBs, an analogous approach can also be employed for the preparation of Li metal anodes. For example, an oriented Cu framework structure as an anode skeleton for Li deposition can be prepared by extrusion‐based DIW 3D printing (Figure 10c).^[^ ^108^ ^]^ The interconnected 3D structure reduces the local current density and can accommodate volume changes during cycling. More importantly, electrochemically active sites on the surface of the Cu skeleton filaments induce lateral Li deposition and growth of Li within the pores parallel to the separator, which effectively prevents the formation of Li dendrites and piercing of the separator. As a result, the Li deposition morphology remains compact even after deposition at a current density of 1 mA cm^−2^ for 20 h. Based on the same working principle, array, and lattice‐type oriented structural skeletons have been fabricated by 3D printing of highly concentrated MXene Ti_3_C_2_T_x_ inks (where T_x_: O, OH, F) and used as anodes after pre‐deposition of 20 mA h cm^−2^ Li metal. The array and lattice structures not only homogenized the Li^+^ flow but also led to preferential Li deposition toward printed filament intervals using the edge‐enhanced electric field tip effect until the dense filling was reached (Figure 10d).^[^ ^47^ ^]^

Besides the usage in anodes, the application of 3D printing also minimizes the trade‐off between high energy and power density in cathodes. As shown in Figure 10e, an ultrathick 3D LiFePO_4_ cathode (1500 µm) printed in a proposed pattern with a high areal capacity of 7.5 mA h cm^−2^ achieves superior electrochemical performance over conventional thick flat electrodes, as its oriented vertical structures reduce both electrode tortuosity and ion/electron transport distance.^[^ ^109^ ^]^

Furthermore, 3D printing can also be utilized for the oriented structural modification of SSE. To overcome localized hotspots on the Li metal surface caused by the poor heat dissipation of the polymer electrolyte and the adverse effects on Li deposition behavior and safety performance,^[^ ^110^ ^]^ Zheng et al.^[^ ^111^ ^]^ compounded BN into PEO‐PVDF polymer electrolytes to improve the performance of all‐solid‐state Li–S batteries. They observed that the added BN enables homogeneous Li stripping/deposition and reversible cathode reaction by accelerating the thermal response and enhancing the mechanical strength of the SSE. In addition, Cheng et al.^[^ ^84^ ^]^ developed a composite electrolyte by incorporating a thermally conductive additive BN into the polyethylene oxide (PEO), where an oriented microstructure of the added BN was realized by 3D DIW printing (Figure 10f). It was shown by microthermal imaging that the maximum surface temperature of the PEO polymer containing oriented BN is lower, which indicates that the heat generated can be effectively diffused from the hot spot to the surrounding area (BN in‐plane direction). As a result, Li deposition is more homogeneous and denser at interfaces under uniform heat distribution, and batteries assembled with PEO/oriented BN composite SSE exhibit higher capacity retention after long cycling.

### 3.2 Template‐Derived Oriented Structure in LMBs

**Figure 11:**
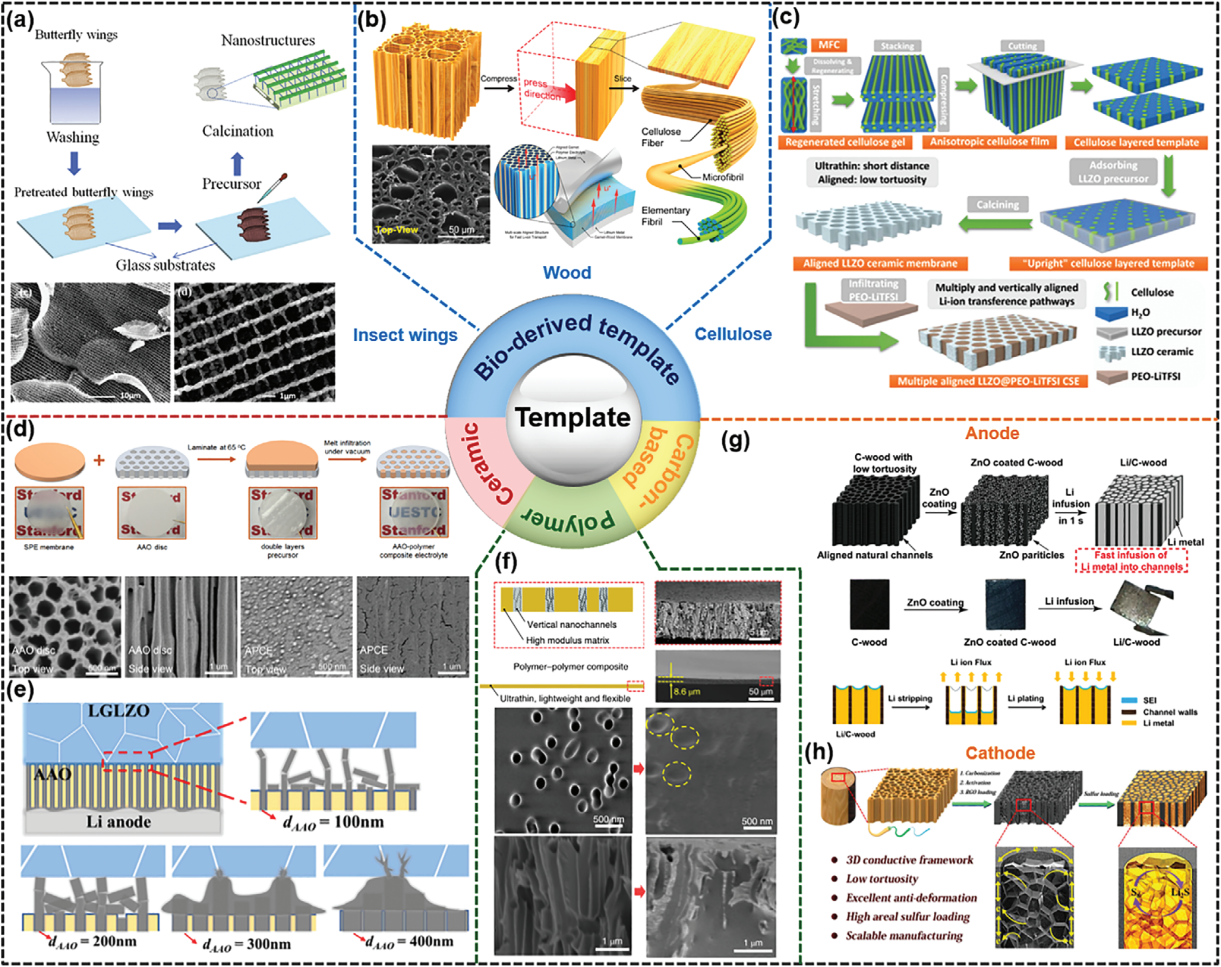
Template‐derived oriented structure in LMBs. Bio‐derived templates fabricated from a) insect wings (Reproduced with permission.^[^ ^112^ ^]^ Copyright 2017, Elsevier), b) wood (Reproduced with permission.^[^ ^69^ ^]^ Copyright 2019, American Chemical Society), and c) cellulose (Reproduced under terms of the CC‐BY license.^[^ ^68^ ^]^ Copyright 2022, John Wiley & Sons Australia, Ltd.). Anodic aluminum oxide (AAO) ceramic templates for d) skeletons of PEO SSE (Reproduced with permission.^[^ ^70^ ^]^ Copyright 2018, American Chemical Society) and e) SSE/Li anode interfacial layer (Reproduced with permission.^[^ ^114^ ^]^ Copyright 2023, Elsevier). f) Polyimide templates with vertically oriented channels for SSE. (Reproduced with permission.^[^ ^64^ ^]^ Copyright 2019, Springer Nature). Carbonized wood with vertically oriented channels for g) Li metal anode skeletons (Reproduced with permission.^[^ ^51^ ^]^) and h) cathode skeletons (Reproduced with permission.^[^ ^88^ ^]^ Copyright 2017, American Chemical Society).

The utilization of oriented structural templates as backbones of the SSE and electrode remains one of the main techniques to reduce tortuosity and improve electrochemical performance. As shown in **Figure** **11**, numerous classes of templates have been used, including naturally derived oriented structural skeletons (wood,^[^ ^69^ ^]^ cellulose,^[^ ^68^ ^]^ insect wings,^[^ ^112^ ^]^ shellfish^[^ ^113^ ^]^), artificial ceramics,^[^ ^70^, ^114^ ^]^ polymers,^[^ ^64^ ^]^ or carbon‐based frameworks.^[^ ^51^, ^88^, ^94^ ^]^

Among the naturally obtained templates, carbonized butterfly wings might constitute ideal natural skeletons for synthesizing 3D electrodes Li_4_Ti_5_O_12_‐TiO_2_ (Figure 11a), as they provide continuous channels and high surface area to enhance the Li^+^ insertion/de‐insertion process and electron transport. This electrode matched with the Li anode can exhibit a high reversible capacity of 169 mA h g^−1^ with ≈100% capacity retention over 80 cycles.^[^ ^112^ ^]^ The use of natural wood, although it was first used in low‐cost sodium‐ion batteries,^[^ ^115^ ^]^ has gradually gained popularity also in LMB research. Figure 11b shows that wood templates with neatly aligned channels were obtained through a sequence of chemical treatment, compression, and slicing. After complete penetration of the precursor solution of LLZO garnet solid electrolyte into this treated wood, LLZO membranes were generated by calcination and subsequent filling with PEO polymer electrolyte.^[^ ^69^ ^]^ Such low tortuosity composite electrolytes with vertical ion channels promote Li^+^ movement through the PEO, PEO/LLZO interface, and the LLZO substrate, resulting in a room temperature conductivity of the composite membrane of up to 1.8 × 10^−4^ S cm^−1^. Afterward, a more facile and practical “stretching–stacking–compressing–cutting” process was introduced by Ni et al.^[^ ^68^ ^]^ by constructing a bio‐template with aligned cellulose fibers. By the infiltration–calcination‐compounding process, low tortuosity LLZO/PEO composite electrolytes were derived as shown in Figure 11c. Benefiting from the same oriented ionic transport pathways, Li^+^ conductivity has been further enhanced to 2.1 × 10^−4^ S cm^−1^ at room temperature.

Beyond naturally obtained templates, artificial skeletons such as ceramic anodic aluminum oxide (AAO) can also compound with electrode/electrolyte. As shown in Figure 11d, Cui et al.^[^ ^70^ ^]^ combined a surface‐modified AAO template with PEO polymer via melt infiltration to fabricate a low tortuosity SSE composite. It was experimentally demonstrated that the ionic conductivity of the AAO/polymer interface is higher than that of just the polymer alone, resulting in a total ionic conductivity as high as 5.82 × 10^−4^ S cm^−1^. Zhou et al.^[^ ^114^ ^]^ introduced an AAO template with vertically aligned nanoscale channels at the garnet electrolyte/Li anode interface to regulate Li deposition behavior and accommodate volume changes (Figure 11e). The in situ reaction of AAO with deposited Li forms lithium aluminate, which further strengthens the confinement function of AAO for deposited Li. AAO with a 300 nm pore has been reported to be the most favorable for delaying the filling rate of Li metal in the pores and inducing uniform Li deposition at the AAO/SSE interface through the pore interconnections, in accordance with findings in an earlier study by Wang et al.^[^ ^58^ ^]^ In addition, polyimide polymer membranes with oriented channels can be used as a substrate to form oriented composite polymer electrolytes with PEO (Figure 11f).^[^ ^64^ ^]^ The ionic conductivity of the infused PEO polymer electrolyte can be enhanced to 2.3 × 10^−4^ S cm^−1^ at 30 °C due to the vertically oriented channels. Finally, carbon‐based templates are widely used as electrode skeletons due to their high electronic conductivity, lightweight, and environmental friendliness. Zhang et al.^[^ ^51^ ^]^ developed a low tortuosity Li–C wood composite anode by infusing molten Li metal into a carbonized wood template with lithophilic ZnO on its surface (Figure 11g). During the stripping/plating process, the Li metal is contained in the oriented channels that inhibit volume changes, thus achieving long‐term cyclic stability. The utilization of carbonized wood filled with reduced graphene oxide can also enable the formation of a 3D current collector with low tortuosity that exhibits high electronic conductivity and high structural stability (Figure 11h).^[^ ^88^ ^]^ When combined with a PEO composite electrolyte, it provides a high areal capacity of 15.2 mA h cm^−2^ for thick sulfur cathodes with a high mass loading of 21.3 mg cm^−2^.

### 3.3 Cold/Heat Treatment for Oriented Structure in LMBs

**Figure 12:**
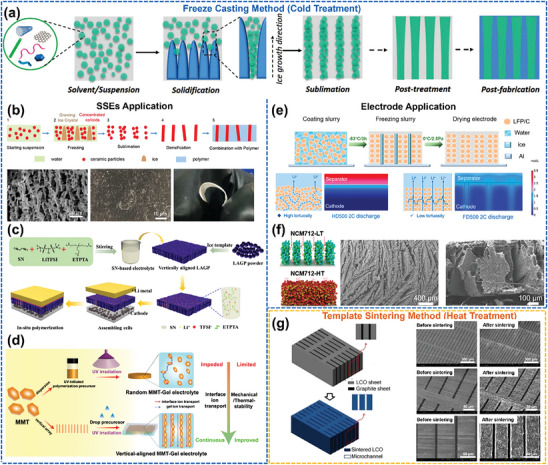
Cool/Heat treatment for oriented structure in LMBs. a) Schematic diagram of the process of the freeze casting method. (Reproduced with permission.^[^ ^116^ ^]^ Copyright 2020, Wiley‐VCH). b) The preparation process and SEM images of the composite PEO SSE with aligned LAGP ceramic skeletons prepared through freeze casting. (Reproduced with permission.^[^ ^74^ ^]^ Copyright 2017, American Chemical Society). c) The preparation process of the composite succinonitrile‐based SSE (Reproduced with permission.^[^ ^73^ ^]^ Copyright 2021, Elsevier). d) The fabrication process of SSEs with vertical‐aligned montmorillonite skeleton (Reproduced under terms of the CC‐BY license.^[^ ^72^ ^]^ Copyright 2022, Springer Nature). Low tortuosity e) LiFePO_4_ (Reproduced with permission.^[^ ^41^ ^]^ Copyright 2021, Elsevier) and f) LiNi_0.7_Co_0.1_Mn_0.2_O_2_ (Reproduced with permission.^[^ ^39^ ^]^ Copyright 2022, Elsevier) cathodes prepared by freeze casting. g) The oriented LiCoO_2_ cathode was obtained by removing the sacrificial graphite green sheets from the solidified composite via sintering at a high temperature. (Reproduced with permission.^[^ ^100^ ^]^ Copyright 2020, Elsevier).

Cold treatment, more accurately referred to as the freeze casting method, is another versatile technique that has been widely used to fabricate porous ceramics, metals, polymers, and carbon nanomaterials with controlled pore orientation. **Figure** **12**a illustrates that freeze casting entails the controlled solidification of a solution/suspension/sol/gel, the sublimation of the solvent (typically water) under reduced pressure, and subsequent densification, resulting in an oriented structural skeleton. Post‐processing, such as infusion, then leads to an oriented composite material.^[^ ^116^ ^]^ The main advantage of this approach is that by micro‐tuning the suspension characteristics and solidification parameters (temperature, velocity, external force field), different pore structures can be produced. Since a variety of aligned porous polymers and polymer‐nanoparticle composites have been demonstrated by freeze‐casting in the early 2000s,^[^ ^117^ ^]^ this technology has gained increasing attention in tandem with the surge in requirements for battery energy density and safety.

For solid‐state electrolyte applications, Yang^[^ ^74^ ^]^ and Wen^[^ ^73^ ^]^ successfully prepared Li_1.5_Al_0.5_Ge_1.5_(PO_4_)_3_ (LAGP) ceramic skeletons with vertically oriented structures by freeze casting, which can reach ionic conductivity of 0.52 × 10^−4^ and 1.17 × 10^−3^ S cm^−1^ (30 °C) when forming composites with PEO (Figure 12b) and succinonitrile (SN) (Figure 12c), respectively. Subsequently, Li et al.^[^ ^72^ ^]^ prepared montmorillonite clay templates with vertically aligned oriented structures by the same method (Figure 12d). Photocuring of ion‐conducting polymers within these templates led to composite electrolytes with fast ion transport properties (1.08 × 10^−3^ S cm^−1^ at room temperature). For electrode applications, a low tortuosity cathode with LiFePO_4_ areal capacity as high as 99.6 mg cm^−2^ can be prepared by freeze casting (Figure 12e).^[^ ^41^ ^]^ COMSOL simulation shows that this oriented cathode structure reduces the concentration polarization by shortening the effective Li^+^ transport pathway and accelerating transport kinetics, leading to improved rate capability. Later, the same research group replaced the active material with a high nickel material LiNi_0.7_Co_0.1_Mn_0.2_O_2_ to obtain a low tortuosity and high capacity cathode, which achieved homogeneous electrochemical reactions and reduced local stress by suppressing the concentration polarization (Figure 12f).^[^ ^39^ ^]^ As a result, the thicker NCM712 electrode with 25 mg cm^−2^ area loading retained 100% capacity after 150 cycles when cycled in the voltage range of 3–4.3 V.

Materials with oriented structures can also be obtained by removing the sacrificial substrate material from the solidified composite via sintering at a high temperature. For instance, Jeong et al.^[^ ^100^ ^]^ fabricated a 3D thick cathode with periodically aligned fast Li^+^ transport channels by stacking LiCoO_2_/graphite sheets and co‐sintering them as sketched in Figure 12g. Although the microchannels of all the cathodes show uniform intervals, the density of open pores decreases gradually with increasing sintering temperature (19% at 975 °C, 7.7% at 1000 °C, 3.4% at 1025 °C), leading to deterioration of the rate performance. An optimized porous 3D cathode with a thickness of 630 µm obtained by sintering at 975 °C achieves a high areal capacity of 31 mA h cm^−2^. Additives such as NaHCO_3_^[^ ^118^ ^]^ and NH_4_HCO_3_^[^ ^97^ ^]^ can be removed from electrode slurries by decomposing them at moderately high temperatures (cf. Figure 9b in Section 2.2). This generates bubbles to form internal fast‐oriented Li^+^ migration channels during the electrode drying process, which greatly reduces the electrode tortuosity and has a negligible effect on the volumetric energy density of the battery.

### 3.4 Electric/Magnetic Field‐Assisted Preparation for Oriented Structure in LMBs

To optimize the structure of battery components, using contactless forces such as electric or magnetic fields is another straightforward and facile approach. However, its applicability is constrained to materials that respond to applied external magnetic fields or electric fields. These materials can be used as sacrificial templates or functional additives to form oriented structures under external electric or magnetic fields.

**Figure 13:**
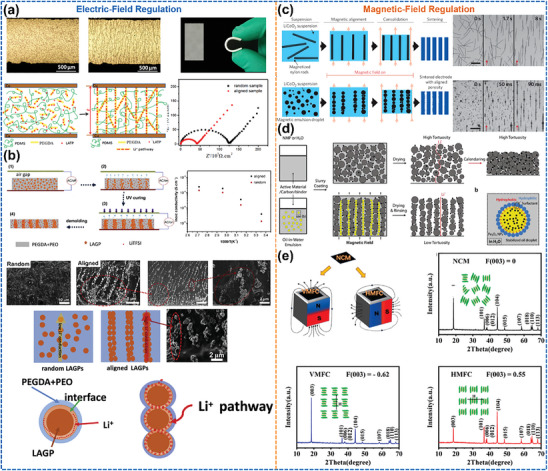
Electric/Magnetic field‐assisted preparation for oriented structure in LMBs. SSEs with a) aligned LATP (Reproduced with permission.^[^ ^119^ ^]^ Copyright 2018, American Chemical Society) and b) LAGP (Reproduced with permission.^[^ ^120^ ^]^ Copyright 2020, Elsevier) particles prepared by applying external electric‐field intensity. c) LiCoO_2_ cathode with directional pore arrays by magnetic field modulation of the magnetized nylon rods (Reproduced with permission.^[^ ^121^ ^]^ Copyright 2016, Springer Nature) and d) stabilized magnetite‐Fe_3_O_4_ nanoparticles‐containing oil droplets. (Reproduced with permission.^[^ ^37^ ^]^ Copyright 2018, Wiley‐VCH). e) NCM622 single crystals aligned along the crystallographic *c*‐axis parallel to the applied magnetic field. (Reproduced with permission.^[^ ^30^ ^]^ Copyright 2022, Elsevier).

As an example, Liu et al.^[^ ^119^ ^]^ prepared a 1 mm thick membrane with aligned Li_1.3_Al_0.3_Ti_1.7_(PO_4_)_3_ (LATP) particles by applying external electric fields of 200–1000 V mm^−1^, under 100 Hz oscillating frequency (**Figure** **13**a). The orientation of the LATP particles was then fixed by thermal/photocuring of the host polymer, resulting in salt‐free ceramic–polymer electrolytes with Li^+^ conductivity up to 2.4 × 10^−6^ S cm^−1^. Using the same method of applying an external 800 V AC field in the vertical direction, particles of Li_1+x_Al_x_Ge_2−x_(PO_4_)_3_ (LAGP) can be aligned in composite polymer electrolytes.^[^ ^120^ ^]^ In this case, the ionic conductivity of membranes with internally *Z*‐aligned and connected LAGP fillers is increased by a factor of 2.7 in the *Z*‐direction (see Figure 13b). The aligned nanoparticles were immobilized in the final film by combining solvent evaporation and photocuring. As shown in the SEM images, aligned and non‐agglomerated LAGP nanoparticles are separated by a thin polymer layer from each other, which offers a fast conduction channel. Combined with the reducing effect of oriented LAGP nanoparticles chain on the crystallinity of the polymer in their vicinity, this may further enhance the ionic conductivity.

Magnetic field regulation is another convenient tool to orient particles within magnetic materials, so it was simultaneously proposed to obtain LiCoO_2_ cathodes^[^ ^121^ ^]^ and graphite anodes^[^ ^122^ ^]^ with directional pore arrays by magnetic field modulation in 2016. As shown in Figure 13c, magnetic microrods and magnetic emulsion droplets are controlled by a magnetic field to realize an oriented distribution in the cathode suspension. After evaporation of the solvent, a thick cathode with aligned pores can be obtained by removing the magnetic microrods and magnetic emulsion through high‐temperature sintering and rinsing with kerosene, respectively. The oriented pore arrays enable fast charge transfer kinetics despite increased cathode thickness, resulting in a more than threefold enhancement of the areal capacity of the electrode.

Thereafter, the same team prepared electrodes with thicknesses exceeding 400 µm with an area capacity of up to 14 mA h cm^−2^ by a second method (Figure 13d).^[^ ^37^ ^]^ Oil droplets containing magnetite Fe_3_O_4_ nanoparticles are stabilized by surfactants at the oil–water interface and thus their orientation can be controlled by a magnetic field, suggesting that stably dispersed magnetic sacrificial additives are critical for obtaining oriented structures. Subsequently, researchers have proposed that single‐crystalline cathode materials (LiFePO_4_,^[^ ^123^ ^]^ NCM523^[^ ^124^ ^]^ and NCM622^[^ ^30^ ^]^) can be preferentially oriented under an external magnetic field, resulting in a neat alignment and a consequent increase in capacity retention and rate performance. As shown in Figure 13e, the extent of parallel alignment of the *c*‐axes with respect to the current collector is estimated using a modified “Lotgering factor” (LF), which in turn yields the effect of the magnetic field on the perpendicular orientation of the (003) crystal plane. Here, LF may be understood as a simplistic estimate of the March–Dollase factor *R* (discussed in section 2.1.5) based on the intensity variation of a single Bragg peak rather than the intensity variations of the entire powder diffraction pattern. The results show that hexagonal single‐crystalline NCM622 particles can be aligned along the crystallographic *c*‐axis parallel to the applied magnetic field at only 0.4 T.

### 3.5 LMBs with Oriented Structures by Electrospinning

Electrospinning is a nanofiber preparation technique that utilizes a high voltage to overcome the surface tension of a solution or melt containing polymers or nanoparticles, causing them to be ejected from the tip of a needle or a nozzle, which then undergoes a process of “drift–stretch consolidation” to form a continuous nanofibrous membrane on a charged plate or collector.^[^ ^125^, ^126^, ^127^, ^128^ ^]^ Key advantages of electrospinning technology are as follows: 1) Nanofibers with manageable diameter range and controllable orientation can be fabricated; 2) the method is applicable to a wide range of materials, such as polymers, metal oxides, carbon nanotubes and so on; 3) the preparation is simple and economical, thus continuous and stable mass production can be achieved. Electrospinning technology for the preparation of novel separators, electrode/SSE skeleton, interfacial functional layer, and other crucial battery components with oriented structure can improve battery performance and safety. In recent years the development of near‐field electrospinning (NFES) has further improved the capability to achieve position‐controlled deposition of nanofibers, significantly expanding the range of applications.^[^ ^129^ ^]^

**Figure 14:**
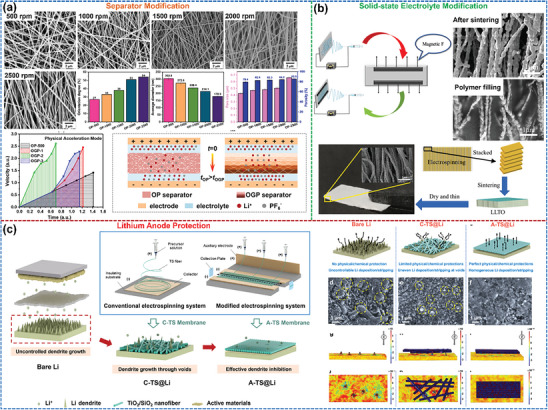
Electrospinning method for oriented structures in LMBs. a) Polyacrylonitrile separators with different orientation degrees and their properties are prepared at different collector speeds. Migration velocity of Li^+^ in separators of different structures and schematic diagram of the mechanism. (Reproduced with permission.^[^ ^62^ ^]^ Copyright 2023, Elsevier) b) SSE with vertically aligned ceramic substrates LLTO prepared by electrospinning technique combined with magnetic regulation. (Reproduced with permission.^[^ ^71^ ^]^ Copyright 2022, Elsevier) c) Aligned lithiophilic TiO_2_/SiO_2_ electrospun nanofiber membrane as an interface layer for uniform Li deposition. COMSOL simulation results of electric field distribution and SEM images of the deposited Li. (Reproduced with permission.^[^ ^130^ ^]^ Copyright 2022, Elsevier).

For separator modification, Xing et al.^[^ ^62^ ^]^ developed a polyacrylonitrile nanofibrous membrane with an orientation gradient along the thickness direction by a continuous electrospinning process with different collector rotation speeds. As can be seen from **Figure** **14**a, the fiber orientation and porosity increase gradually with the acceleration of collector rotation speed. The separators prepared by sequentially varying the rotational speeds in the sequences 500–1000–1500–2000–2500, 2500–1500–500–1500–2500, and 500–1500–2500–1500–500 rpm are labeled as OGP‐1, OGP‐2, and OGP‐3 respectively. Among these, the OGP‐2 separator maintains a high porosity of ≈90%, ideal electrolyte wettability, and thermal stability, while improving the tensile strength by a factor of 2.5 compared to the OP‐500 separator (fabricated with constant 500 rpm collector rotating speeds). Compared with disordered separators, the OGP‐2 separator has an orientation gradient in the direction of ion movement, which provides an optimal transport path for Li^+^ to swiftly move between electrodes.

For the modification of SSE, the electrospinning technique is typically combined with an applied external magnetic field, forming oriented SSE substrates by sintering a fibrous membrane electrospun from precursor solutions with added ionic conductive ceramics or precursor salts. Li et al.^[^ ^71^ ^]^ applied an improved electrospinning technique combining magnetic strips with the collector to obtain oriented nanofibers, followed by stacking and sintering to yield vertically aligned and oriented SSE ceramic substrates Li_3x_La_2/3−x_TiO_3_ (LLTO) with an ionic conductivity as high as 4.67 × 10^−4^ S cm^−1^ after combining with PEO (polyethylene oxide)/PEG(Polyethylene glycol) polymers (Figure 14b).

For Li anode protection, Wang et al.^[^ ^130^ ^]^ fabricated a highly aligned lithiophilic TiO_2_/SiO_2_ (A‐TS) electrospun nanofiber membrane with high binding energies to Li (Figure 14c). The high binding energy of A‐TS to Li attracts more dead Li to react with the SiO_2_, thus avoiding uncontrolled deposition on the anode surface. Besides, the oriented structure can homogenize the Li^+^ diffusion flow to inhibit Li dendrite formation. COMSOL Multiphysics software was performed to investigate the effect of the oriented structure on the electric distribution after Li dendrite formation. The SEM images and simulation results show that the A‐TS membrane uniformly distributes the electric field, which can effectively weaken the tip effect to inhibit the growth of Li dendrites.

### 3.6 Laser Modification for Oriented Structure in LMBs

Laser technology is particularly suitable for cutting, etching, and punching processes in the production of battery components due to its ability to achieve high‐precision processing and manufacturing. Through laser processing, oriented structured electrodes and SSE with precise dimensions and shapes can be prepared. However, it is worth noting that some materials are highly sensitive to laser radiation, and are easily damaged or degraded during laser processing. Therefore, the relationship between material damage and performance enhancement needs to be carefully weighed when applying laser technology to battery manufacturing. In addition, removing impurities such as particles from laser cutting to prevent them from deteriorating cycle life and safety of the produced batteries.

**Figure 15:**
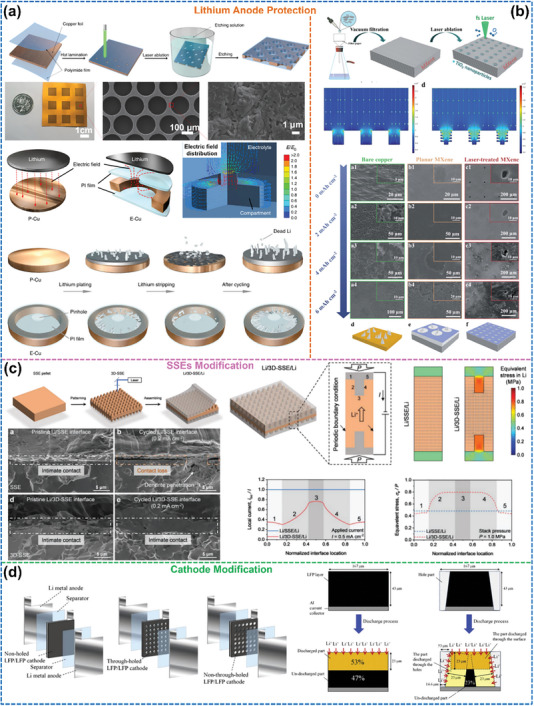
Laser technique for oriented structure in LMBs. a) The preparation process and SEM images of Cu foil with the oriented microporous array. The simulation results of electric field distribution and schematic diagram of Li dendrite growth behavior. (Reproduced under terms of the CC‐BY license.^[^ ^81^ ^]^ Copyright 2018, Springer Nature) b) Preparation process of MXene membranes with oriented microchannels. The simulation results of electric field distribution and SEM images of the Li deposition morphology. (Reproduced with permission.^[^ ^131^ ^]^ Copyright 2020, Royal Society of Chemistry) c) Preparation process of the 3D‐SSE with surface‐oriented patterning and the simulation results of the stress/current distribution at the Li/SSEs interface. SEM images of the Li/SSE interface morphology before and after cycling show that the Li/3D SSE interface stays tighter. (Reproduced with permission.^[^ ^66^ ^]^ Copyright 2021, Wiley‐VCH) d) Schematic diagram of the cells with non‐holed, through‐holed and non‐through‐holed cathodes. Schematic illustration of the cathode discharge region and the mechanism of enhanced active material utilization for non‐holed and through‐holed LFP/LFP cathodes. (Reproduced with permission.^[^ ^132^ ^]^ Copyright 2019, Elsevier).

For the protection of LMAs, Zou et al.^[^ ^81^ ^]^ first proposed a laser drilling/alkaline etching process to develop unique Cu collectors with oriented microporous array structures, aiming to guide Li dendrite growth in specific directions and reduce the risk of short circuits within the battery (**Figure** **15**a). Unlike the traditional concept of suppressing Li dendrites, the simulation results demonstrate that the electric field inside the micropores is deflected from the vertical to the horizontal direction, thus inducing the Li dendrites to grow “horizontally,” i.e., parallel to the separator, prolonging the lifetime of the LMBs even in the extreme case of massive Li dendrites growth. Subsequently, Xiong et al.^[^ ^131^ ^]^ employed laser ablation to create oriented microchannels in MXene membranes to modify the electric field distribution (Figure 15b). The simulation results reveal that due to the inherent layer‐by‐layer property of MXene, many nanoscale edges within the channels exacerbate the tip effect to induce the uniform Li^+^ flux toward the channel interior. The SEM image shows that as the deposition capacity increases, the Li metal remains evenly confined within the oriented channels without exhibiting dendritic morphology.

In addition, laser processing techniques for the fabrication of oriented structures have already been employed in the fabrication of orientationally ordered SSEs. Xu et al.^[^ ^66^ ^]^ applied high‐precision laser cutting to obtain 3D‐SSE (Li_6.4_La_3_Zr_1.4_Ta_0.6_O_12_, LLZTO) with surface‐oriented patterning (Figure 15c). From an electrochemical perspective, the increase in the effective contact area of 3D‐SSE with Li reduces the local current density, thus delaying the Li deposition/stripping at the interface. From a mechanical viewpoint, the presence of 3D‐oriented patterned structures can induce a highly deviatoric stress state of the Li metal near the interface under limited stack pressure, allowing region 3 (in the right‐hand side graphs of Figure 15c) to generate the highest stress at a relatively high current density and thus promoting Li creep. Therefore, the final interfacial contact and morphology remain favorable after long cycling.

The laser processing technique has also been widely used in cathode processing to obtain an internally oriented structure that improves the electrolyte wettability and reduces the tortuosity, maintaining a superior rate performance even at high active material surface loadings.^[^ ^98^, ^132^, ^133^, ^134^, ^135^, ^136^, ^137^, ^138^ ^]^ As shown in Figure 15d, Tsuda et al.^[^ ^132^ ^]^ developed a laser system that can form 20 000 oriented holes on the cathode surface in one second. In both “through‐hole” and “non‐through‐hole” cathodes, Li^+^ can be inserted/de‐inserted more efficiently through the holes formed by a pico‐second pulsed laser, leading to a shorter Li^+^ transport pathway and maintaining a high‐rate performance in thick cathodes. As a result, the active material utilization (the percentage of the areas of the discharged parts) can be increased from 53% to 77% at a 10C rate.

### 3.7 Other Modification Methods for Oriented Structure in LMBs

**Figure 16:**
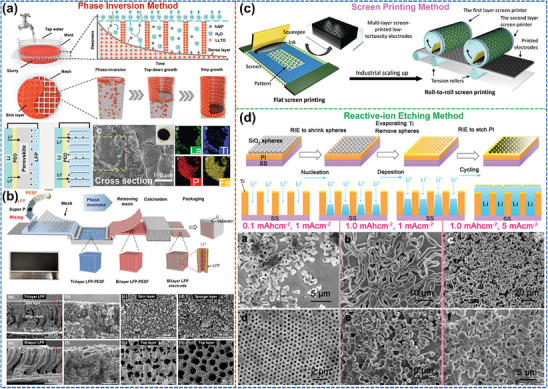
Other techniques for oriented structure in LMBs. a) The preparation process and SEM images of the LLZO skeleton in SSEs (Reproduced with permission.^[^ ^140^ ^]^ Copyright 2018, Wiley‐VCH) and b) LiFePO_4_ cathode (Reproduced with permission.^[^ ^141^ ^]^ Copyright 2021, American Chemical Society) with oriented pore channels prepared by phase inversion method. c) LiNi_0.6_Mn_0.2_Co_0.2_O_2_ cathodes with controllable pattern/diameter/density of channels prepared via screen printing method. (Reproduced with permission.^[^ ^143^ ^]^ Wiley‐VCH) d) Preparation process of the polyimide polymer layer on Li anode with high‐aspect‐ratio vertical nanoscale channels and the SEM images of deposited Li. (Reproduced with permission.^[^ ^52^ ^]^ Copyright 2016, American Chemical Society).

Apart from the six widely explored main methods discussed above, there are also some emerging alternative methods for the fabrication of oriented structural components in LMBs. Among these, the phase inversion method allows for the preparation of porous materials by adding a non‐solvent to the homogeneous solution system or changing the temperature, resulting in the formation of a solid phase of the material that was originally dissolved.^[^ ^139^ ^]^ The solid phase gradually assembles to form membranes, fibers, or other desired shapes during the phase inversion process. In combination with an aligned template, oriented porous structures with different porosity and pore size distributions can be achieved. Wang et al.^[^ ^140^ ^]^ used Nylon mesh as a template and obtained a Li_0.34_La_0.51_TiO_3_ (LLTO) framework with oriented pores (**Figure** **16**a). Due to the solubility of the poly(ether sulfone) polymer in N‐methyl‐2pyrrolidone (NMP) solvent and insolubility in water, rapid convection between water and NMP induces the formation of polymer microchannels as nascent pores. From the battery structure diagram and the elemental distribution mapping, the 2D contact between the cathode material and the SSE is transformed into a 3D contact, and the Li^+^ migration path is shortened. Likewise, Yu et al.^[^ ^141^ ^]^ developed vertically aligned microchannels in the cathode by the same method, with a stainless‐steel mesh instead of the Nylon template (Figure 16b). SEM images show that long and uniform microchannels are formed, thus significantly reducing the electrode tortuosity to 1.62. Therefore, a 1.2 mm ultrathick LiFePO_4_ cathode with an active material areal capacity of up to 100 mg cm^−2^ can still deliver superior electrochemical performance.

Moreover, screen printing to prepare oriented structure battery components is economical and suitable for mass production.^[^ ^142^, ^143^ ^]^ The slurry is spread on the screen‐printing template and scraped into its holes with a blade to form a pattern. The structure of the printing template and the size of the holes then influence the orientation structure. A final drying and curing process stabilizes the bonding of the slurry to the substrate. As shown in Figure 16c, LiNi_0.6_Mn_0.2_Co_0.2_O_2_ cathodes with controlled pattern/diameter/density of channels were prepared via roll‐to‐roll screen printing method by Zhu et al.^[^ ^143^ ^]^ Compared with the specific capacity of a traditional cathode prepared by bar‐coating (10 mA h g^−1^), this electrode still can maintain a high 72 mA h g^−1^ at a 6C cycling rate due to the low tortuosity.

Reactive‐ion etching (RIE) is used in the preparation of microfabricated structures, where atoms or molecules on the surface of the material can be removed by ion bombardment through a chemical reaction from energetic ions and reactive gases in a vacuum. Cui et al.^[^ ^52^ ^]^ innovatively applied this method to prepare functional electrode coatings with oriented structures to inhibit the growth of Li dendrites (Figure 16d). The polyimide polymer coating with high‐aspect‐ratio vertical nanoscale channels homogenizes the Li^+^ flow on the electrode surface and contributes to the formation of uniform nucleation sites. As the porous polyimide coatings are electrically insulating, the deposited Li is confined within its nano‐channels rather than on the surface. SEM images show that the Li deposition still grows in an island morphology without exhibiting dendritic morphology.

### 3.8 Comparison of Methods for Preparation of Oriented Components in LMBs

**Figure 17:**
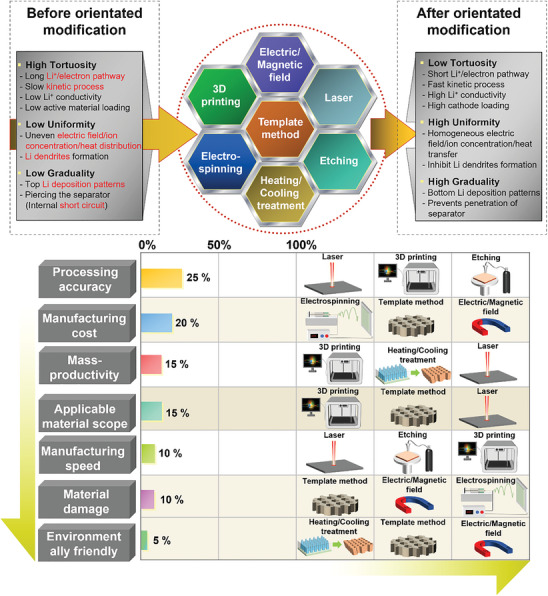
Comparison of Methods for Preparation of Oriented Components in LMBs.

As shown in **Figure** **17**, by introducing an orientation assessment system that includes factors such as tortuosity, homogeneity, and property graduality, the most suitable oriented structural modification method can be achieved. The optimization of the orientation degree helps to find a balance between the energy density, rate capability, and safety performance of the LMBs.

To develop a comprehensive evaluation scheme and select the most suitable method for preparing oriented structures for practical LMBs in fabrication, it is necessary to weigh seven factors: processing accuracy, cost, mass‐producibility, material applicability, processing speed, the degree of damage to the material, and environmental friendliness. For each of these different indicators, Figure 17 indicates the top three most advantageous processing technologies. Processing accuracy, manufacturing costs, and mass‐productivity are the highest priority factors to be evaluated, with processing accuracy being the most critical to ensure the effectiveness of the application. 3D printing, laser processing, and etching thus appear to be advantageous. Laser processing can achieve high precision, usually at the micron or submicron size level.^[^ ^144^ ^]^ The precision of 3D printing depends on the printing technology and equipment, and can generally reach a precision of tens to hundreds of microns.^[^ ^145^, ^146^ ^]^ High‐resolution 3D printing technologies, such as light‐curing 3D printing, can achieve a much smaller size range. The precision of etching also depends on process parameters and equipment, and a similar range of precision to laser processing and 3D printing can often be achieved at reduced cost.

To further improve the efficiency and accuracy of battery manufacturing technologies and to reduce the cost, it is also essential to optimize the preparation techniques as well as the characterization and testing methods. For example, combining oriented structure preparation techniques with nondestructive, accurate, and fast electrode microcharacterization methods, such as micro‐X‐ray computed tomography (nano XCT^[^ ^147^ ^]^), enables timely adjustments to the process parameters and lays the foundation for subsequent process simulation and optimization studies. Besides, time‐consuming and labor‐intensive exhaustive experimental process testing may be phased out with the progress in digital simulation and digital twin technology to accelerate the determination of optimal process strategies and process parameters in the development of new and optimization of existing battery manufacturing processes.^[^ ^148^, ^149^ ^]^ Using technologies such as big data and artificial intelligence, real‐world battery products, and preparation systems/processes will be mapped into virtual space, and large amounts of real‐time data on production and manufacturing can be accessed through sensors and the Internet of Things, so as to realize the exchange of information and synergy between the entity and the virtual body to guide researchers. Through mathematical modeling and analysis of the battery preparation system or process, the operation of the preparation system can be simulated using computer software to predict, evaluate and optimize the system performance.

## 4 Mechanisms by which Oriented Structures Enhance Battery Performance

### 4.1 Oriented Structures for Boosting Energy Density

**Figure 18:**
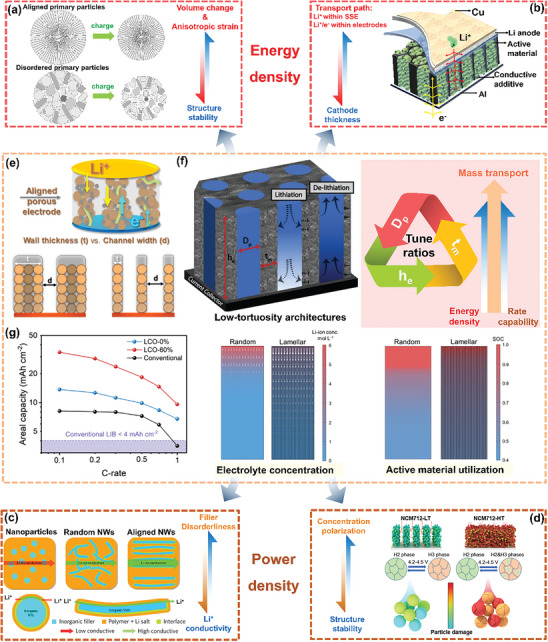
Mechanisms for increasing practical energy density and rate capability of LMBs using oriented structure optimization. a) Reducing volume change and anisotropic strain inside the active materials to inhibit the microcracks formation. b) Reducing Li^+^ migration path through decreasing the tortuosity of electrodes. c) Formation of fast ion migration channels on the surface of the oriented fillers. (Reproduced with permission.^[^ ^91^ ^]^ Copyright 2017, Springer Nature) d) Suppressing the concentration polarization and ensuring fast Li^+^ transport kinetics to improve the structural stability of the active materials. (Reproduced with permission.^[^ ^39^ ^]^ Copyright 2022, Elsevier) e) Wall thickness (t) and channel width (d) significantly affect electrochemical kinetics in thick porous electrodes. (Reproduced with permission.^[^ ^154^ ^]^ Copyright 2021, American Chemical Society) f) Oriented structure modification methods require the regulation and trade‐off of parameters, such as the pore diameter *D*_p_, total electrode height *h*_e,_ and thickness of the skeleton matrix *t*_m_ in the oriented electrodes should reach the optimal ratio to combine high energy density and rate capability. (Reproduced with permission.^[^ ^155^ ^]^ Copyright 2022, Wiley‐VCH) g) The areal capacity and simulation results of vertically lamellar electrodes prepared by bidirectional freeze‐casting and compression‐induced densification method. (Reproduced with Permission^[^ ^156^ ^]^).

In conventional batteries, increasing the thickness of the electrode to enhance the active material loading is one of the ways to increase the energy density of LMBs. However, an increase in the thickness of the electrode layer is inevitably accompanied by a corresponding increase in Li^+^/electron transport path lengths and typically causes electrolyte wetting problems, so that only a portion of the active material remains accessible to undergo redox reactions. Both effects will reduce the practical energy density.^[^ ^132^, ^135^ ^]^ In order to enable the use of thick cathodes for battery application, it is thus imperative to apply oriented structural modifications to retain a stable ionic/charge transfer resistance per unit electrolyte/active surface area that does not change substantially with increasing electrode thickness. Specific mechanisms to achieve this target include:

1. 3D‐oriented structure modifications that increase the active material/electrolyte contact area (active surface area) and enhance the wettability of the electrolyte to ensure a high discharge rate performance of thick layer cathodes over long cycles.^[^ ^136^ ^]^ Since Li^+^ diffusion is much faster through the electrolyte solution within the micrometer‐sized oriented holes than through the layers of the active material; the charge/discharge state of the active material close to the collector is similar to the one of the active material closer to the electrolyte, thus enabling a nearly full utilization of the active material.^[^ ^132^, ^150^ ^]^
2. Preparation of micron‐sized secondary cathode particles consisting of radially aligned single‐crystalline primary particles by microstructure engineering to shorten Li^+^ migration paths, accommodating volume changes and dissipating the internal strain from a microscopic perspective (**Figure** **18**a).^[^ ^31^, ^34^, ^35^, ^36^, ^77^, ^78^ ^]^
3. Construction of oriented conductive skeletons as electrode substrates or oriented non‐conductive skeletons (i.e., polymer electrolyte substrates) to guide Li^+^ movement to preferentially move in a direction perpendicular to the electrode planes and (in the case of the electrolyte) to reduce polymer crystallinity, accelerating and guiding the fast transport of Li^+^/electrons from a macroscopic perspective (Figure 18b).^[^ ^43^, ^79^, ^80^, ^151^ ^]^
4. The construction of low‐tortuosity hosts loaded with high‐efficiency sulfur cathode catalysts (e.g., Al_3_Ni_2_^[^ ^152^ ^]^) or absorbent (e.g., Fe_3_C^[^ ^153^ ^]^) that can promote polysulfide conversion and reduce the solubility of polysulfide intermediates, suppressing the shuttle effect and improving battery capacity retention.^[^ ^9^ ^]^

### 4.2 Oriented Structures for Enhancing Electrode Rate Capability (Power Density)

Orientational structural modifications also have a significant impact on the rate capability of the battery, contributing to the achievement of high‐power output in a short period of time while maintaining a long lifetime. Specific mechanisms include the following:

1. For solid‐state batteries, nanocomposites of ion‐conducting ceramics with polymer substrates are widely considered to synergize the performance, properties, and processing advantages of the two classes of electrolytes. One contributing factor is that the addition of fast ionic conductors reduces the crystallinity of the polymer in the vicinity of the polymer: ceramic interface, which also enhances the solubility of Li^+^ dopant salts and may generate new fast ion migration channels within that interphase. Some authors suggest that functional groups on the surface of the fast‐ion conductors also promote the dissociation of the lithium dopant salt leading to an increase in the local concentration of free Li^+^ within the polymer: ceramic interphase and a higher Li transference number. Notably, the morphology, distribution, and structure of the ion‐conducting ceramic within the polymer have a strong impact on the overall ionic conductivity of the composite polymer solid‐state electrolyte. This can be understood from the fact that a fast dc ionic conductivity requires a percolation of the fast‐conducting interphase regions across the SSE.

As shown in Figure 18c, Cui et al.^[^ ^91^ ^]^ prepared Li_0.33_La_0.557_TiO_3_ (LLTO) nanowires with different alignment orientations (0°±5°: Perpendicular to the electrode, 45°±9°, 90°±8°: Parallel to the electrode) using an electrospinning technique matched with a high‐speed grounded drum collector and form a composite with a PAN/LiClO_4_ polymer electrolyte. They found that the overall ionic conductivity varies with the orientation angle of the nanowires in these composite electrolytes. The overall conductivities of the composite electrolyte with internal nanowire orientation angles of 0°, 45°, and 90° were 5.02 × 10^−5^, 2.24 × 10^−5^, and 1.78 × 10^−7^ S cm^−1^, respectively.

Subsequent replacement of the ionic conductor nanowires with inert ZrO_2_ nanowires resulted in the same phenomenon, thus the authors propose that the interfacial conductivity between Li^+^ conducting nanowires and polymer electrolytes reaches values comparable to the ionic conductivity of liquid electrolytes. For this to translate into a high total conductance, it is important that the fast‐conducting regions are interconnected across the electrolyte layer. In such ceramic‐in‐polymer composite with oriented LLTO nanowires without cross‐junction, multiple fast ion pathways including polymers, ceramics, and polymer/ceramic interphases cooperate to enhance rate performance and thus the achievable cycling rates.

1. Reduced electrode tortuosity suppresses concentration polarization and ensures fast Li^+^ intercalation, deintercalation, transportation, and deposition kinetics, resulting in excellent rate capability (Figure 18d).^[^ ^39^ ^]^ Higher homogeneity of electrochemical reactions in low‐tortuosity thick electrodes facilitates the reduction of anisotropic stresses within the particles for structural integrity, robust electrolyte interfaces, and excellent cyclability under a high cycle rate.

### 4.3 Trade‐Off between Energy Density and Power Density

It is worth noting that the oriented structure modification method requires adjustments of various parameters to maximize both energy density and power density while minimizing the tradeoff between these two factors. The relationship between these two properties can be effectively balanced by constructing a 3D‐oriented pore structure within the electrode. Yu et al.^[^ ^154^ ^]^ suggested that two parameters, wall thickness (*t*) and channel width (*d*) (Figure 18e) significantly affect electrochemical kinetics in thick porous electrodes. In this regard, they prepared LiNi_1/3_Mn_1/3_Co_1/3_O_2_ (NCM111) cathodes (20–25 mg cm^−2^) with various values of *d* and *t* by ice‐templating method and found that at a high rate of 2.5C the specific capacities of the cathodes with *d* = 18.4 µm / *t* = 5 µm were much higher than those of cathodes with *d* = 30.7 µm / *t* = 16 µm, *d* = 9.9 µm/*t* = 22.5 µm and without pore structure.

Werner et al. concluded from computational and experimental results of earlier studies, that the optimum range for the diameter of low‐tortuosity pores and the thickness t_m_ of the solid matrix containing active material should fall within the range of 5–20 µm, as this allows for a simultaneous enhancement of power output and energy density.^[^ ^154^, ^155^ ^]^ Additionally, they stated that the pore diameter (*D*_p_), the total electrode thickness (*h*_e_), and the thickness of the solid skeleton (*t*_m_) need to be tuned precisely in order to overcome the limitations of the mass transport as the key performance‐limiting factors (Figure 18f). Nevertheless, the lithiation process remains the main battery performance limiting factor at higher current density even when using low‐tortuosity oriented electrodes.

Although the rate capability of the battery improves with a decrease in t_m_, it comes at the cost of lower volumetric density. Yu's group then further modified their ice‐templating method by coupling bidirectional freeze‐casting and compression‐induced densification to prepare densified vertically lamellar electrodes, simultaneously achieving high volume/mass–energy density and power density.^[^ ^156^ ^]^ Comparing the areal capacities at various C rates in Figure 18g, the vertically lamellar electrode can reach a maximum areal capacity of ≈33 mA h cm^−2^ (volumetric capacity of ≈300 mA h cm^−3^) and a retained areal capacity of ≈10 mA h cm^−2^ at 1C. Simulation results show that a vertically lamellar electrode provides a more uniform concentration along the electrode thickness and a higher active material utilization across the electrode, leading to a uniform charge state of electrode material and stable cyclability.

### 4.4 Oriented Structures for Safety Optimization

In the late 1980s, the company Moli Energy developed the first‐generation commercial rechargeable LMBs with remarkably higher energy density when compared to LIBs with graphite anodes (theoretical capacity: 372 mA h g^−1^). However, these LMBs were quickly phased out due to serious safety issues. Till today, eliminating the hazard of Li dendrite formation remains a major obstacle for commercial application of LMBs. From a thermodynamic perspective, lowering the surface energy of Li facilitates the formation of a 1D whisker‐like deposition morphology (with a larger Li metal surface compared to flat decomposition), while high energy barriers for ion mobility inhibit the redistribution of Li to surrounding regions; the two main factors promoting the formation of Li dendrites.^[^ ^157^ ^]^ Uneven Li^+^ flow in the various theoretical models is considered the direct cause of the nonuniform distribution of nucleation sites, leading to the formation of Li dendrites.^[^ ^53^, ^57^, ^158^ ^]^ Therefore, the utilization of oriented structural modification to prevent the formation of Li dendrites and retard separator/SSE puncture has become a research hotspot. The mechanisms of inhibiting Li dendrite formation and separator puncture by constructing oriented structures can be divided into two main aspects: 1) ex ante prevention and 2) ex post suppression/regulation.

#### 4.4.1 Ex‐Ante Prevention

Ex‐ante prevention inhibits the formation of Li dendrites by constructing 3D‐oriented structures, which delays the formation of dendritic morphology from the root cause.

##### Oriented Structures for Reducing Local Current Density/Volume Change

The “Sand's time” model for predicting the nucleation and growth of Li dendrites suggests that the ion concentration gradient below a critical current density remains very small, preventing Li dendrite formation. However, the Li^+^ concentration in the vicinity of the anode decreases to zero within Sand's time *t*_Sand_, leading to a sudden increase in the electric field and rapid deposition of nuclei for Li dendrites.^[^ ^159^, ^160^, ^161^ ^]^

(15) $$t_{Sand}=\pi D\frac{{\left(z\;F\;c_{0}\;\right)}^{2}}{4\;{\left(It_{a}\right)}^{2}}$$

where *z* is the charge number of the cation (z = 1 for Li^+^), *c*_0_ is the bulk salt concentration, *F* is the Faraday's constant, *I* is the current density, and *t*_a_ = 1 − *t*_Li_ is the transference numbers of the anions.^[^ ^162^ ^]^ This means that reducing the effective current density *I* will delay the initial growth of Li dendrites. Therefore, constructing 3D‐oriented structured collectors or contouring the Li/SSE interface significantly increases the electrode/electrolyte contact area, thereby reducing the local current density and retarding the formation of dendrites from the source.

**Figure 19:**
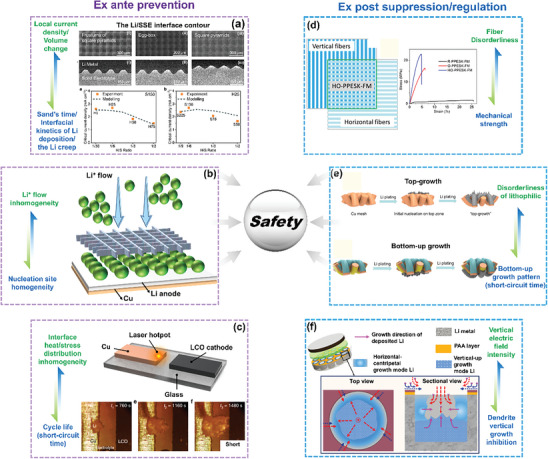
Mechanisms for improving the safety of LMBs using oriented structure improvement. a) Argyrodite solid electrolytes (Li_6_PS_5_Cl) with differently contoured surfaces prepared by 3D printing increase the critical current density. (Reproduced with permission.^[^ ^163^ ^]^ Copyright 2024, Royal Society of Chemistry). b) Oriented structured separator or the artificial interface layer increases the homogeneity of Li nucleation/deposition to inhibit the dendrite growth. c) Localized hot spots at the interface accelerate the growth of Li dendrites. (Reproduced under terms of the CC‐BY license.^[^ ^110^ ^]^ Copyright 2019, Springer Nature) d) Oriented fiber layers are thermally pressed in an ordered stack to enhance mechanical properties. (Reproduced with permission.^[^ ^59^ ^]^ Copyright 2019, Springer Nature) e) Regulating Li growth pattern by modulating the lithiophilic distribution of the deposition skeleton. (Reproduced with permission.^[^ ^164^ ^]^ Copyright 2021, Elsevier) f) 3D vertically oriented holes inside the Li metal anode alter the growth direction of Li dendrites via optimizing the electric/concentration field distribution, ultimately delaying the time of separator puncture. (Reproduced with permission.^[^ ^82^ ^]^ Copyright 2021, Elsevier).

As shown in **Figure** **19**a, Bruce's group^[^ ^163^ ^]^ utilized 3D printing to form argyrodite solid electrolytes (Li_6_PS_5_Cl) with differently contoured surfaces (peak separations S, height profiles H). It was found that the eggbox surface exhibits higher critical currents than frustums of square pyramids and square pyramidal surfaces when considering the current distribution, interfacial kinetics of Li deposition, and the Li creep. Increasing the peak height at a constant peak separation or decreasing the peak separation at a constant peak height both result in a tendency for the critical current to increase and then decrease. However, the charging current for dendrite‐free formation can only be increased by ≈50% compared to flat interfaces, indicating that this method of contouring or otherwise roughening the interface (as discussed earlier in Figure 15c) is of limited effect in addressing safety issues such as dendrite‐induced short circuits.

##### Oriented Structures for Uniform Li^+^ Flow

The Li dendrite formation process is divided into three stages, including the generation of SEI passivation film, nucleation of deposition sites, and growth stage after nucleation.^[^ ^165^ ^]^ By applying oriented structures in LMBs to regulate the distribution of Li^+^ flux, it is expected that uniform Li nucleation and growth can be fundamentally achieved to suppress Li dendrite formation, which enhances the safety performance.^[^ ^52^, ^53^ ^]^ For instance, the uniformity of the separator pores in Figure 19b is positively correlated with the homogeneity of Li nucleation/deposition, implying that enhancing the uniformity of the oriented pores including pore size, density, and distribution will inevitably result in flatter and denser Li deposits.

##### Oriented Structures for Optimizing Interfacial Stress/Heat Distribution

Temperature and stress distribution across the Li anode/electrolyte interface are closely correlated with interfacial mass transfer homogeneity and thus have a critical impact on the formation of Li dendrites.^[^ ^166^ ^]^ As shown in Figure 19c, Cui et al.^[^ ^110^ ^]^ induced a localized hotspot at the interface by laser irradiation, and the locally enhanced surface exchange current density at the hotspot induced a significant growth of Li metal compared to the surrounding region with lower temperatures. At a constant current of 30 µA, the Li metal at the hotspot contacted the LiCoO_2_ cathode and shorted after only 1480 s. Overall, temperature distribution affects the interfacial Li deposition behavior in the following ways: (I) temperature affects the viscosity and crystallinity of the electrolyte, thus affecting the local Li^+^ mobility; (II) temperature affects the dissociation of Li salts, as well as the structure and properties of the SEI; (III) temperature affects the Li nucleation size and density; (IV) temperature affects the mechanical properties and thereby the interfacial contact, such as polymer electrolytes softening at elevated temperatures resulting in a tighter interfacial contact. Thus, as discussed in the context of Figure 10f in Section 3.1, a more uniform interfacial thermal distribution can be achieved by orienting and uniformly distributing the thermally conductive BN in the polymer SSE, resulting in a more uniform and dense Li deposition.^[^ ^84^ ^]^ Similarly, increasing interfacial stress induces the creation of a denser Li deposition morphology and facilitates tight interfacial contact, whereas dendritic morphology proliferates in the absence of stress, which tends to puncture the separator/SSE.

#### 4.4.2 Ex‐Post Suppression/Regulation

Since Li deposition is a complex stochastic process and various modification methods are prone to eventually fail after long‐term cycling due to the damage of introduced material or modified structure, Li dendrites cannot be completely eradicated and may still appear uncontrollably after prolonged cycling. Therefore, ex‐post suppression/regulation is needed, which refers to mitigation techniques that should be used to inhibit piercing the separator/SSE to prevent internal short circuits once Li dendrites have already been nucleated.

##### Oriented Structures for Improved Mechanical Properties

The mechanical properties of the oriented structure are critical to the cycling life of the LMBs, as the major volume change of Li anodes during cycling and dendritic deposition is highly susceptible to causing damage to the electrolyte/Li interface, leading to the failure of LMBs. For example, the use of SSE with high Young's modulus, especially inorganic SSE, acts as a strong barrier against Li dendrite penetration and is considered to be an effective strategy.^[^ ^167^ ^]^ However, the inherent anisotropy in the mechanical properties of the oriented structure leads to significantly different tensile/compressive strengths in the horizontal direction than in the vertical direction. Therefore, the oriented structures can be combined and arranged to improve cell performance without sacrificing mechanical strength. As shown in Figure 19d, Shen et al.^[^ ^59^ ^]^ fabricated oriented poly(phthalazinone ether sulfone ketone) fibrous membranes using an electrostatic spinning high‐speed collector, and then hot‐pressed the two membranes vertically overlapping each other. This fibrous membrane showed a high tensile strength of 22.8 MPa both horizontally and vertically, as well as higher porosity, electrolyte uptake, and ionic conductivity compared to the disordered fibrous separator.

##### Oriented Structures for Optimizing Deposition Patterns

Considering that Li deposition is a coupled diffusion‐reaction process regulated by ion/electron fields, adjusting the lithophilicity and conductivity of the 3D collector has been shown to be effective in modulating the associated deposition patterns.^[^ ^86^ ^]^ As in Figure 7 discussed in Section 2.2.2, Liu et al.^[^ ^164^ ^]^ transformed the Li deposition pattern into a “bottom–up” growth mode by coating the bottom and top of the Cu mesh with lithophilic Au and lithophobic polymer blends, converting the lithiophilic distribution of the 3D Cu skeleton into a vertically oriented gradient property (Figure 19e). Therefore, in the case of uneven Li deposition, it can greatly delay the time for Li dendrites to contact the separator/SSE, thereby delaying the occurrence of internal short circuits and improving the cycle life of the LMBs.

##### Oriented Structures for Regulation of Li Dendrite Growth Direction

Optimizing the growth direction of Li dendrites when they have already been formed can also significantly reduce the risk of internal short circuits. Some researchers have recently focused on reorienting the growth of Li dendrites during Li deposition rather than eliminating them.^[^ ^81^, ^168^, ^169^ ^]^ As shown in Figure 19f, altering the electric/concentration field distribution by constructing 3D vertically oriented holes inside the Li metal anode allows the Li dendrites to grow vertically upward and centripetally at the same time, and ultimately squeeze each other to be densely deposited, which effectively delays the time of separator puncture.^[^ ^82^ ^]^ It is worth noting that although none of these methods can completely eliminate needle‐like/mossy Li dendrites, they can still significantly improve the safety and cycle life of LMBs.

## 5 Conclusion and Outlook

Overall, rationally controlling the orientation degree of individual components in LMBs, including the macro‐arrangement/micro‐crystalline orientation of electrode active materials, separator fibers/pores, SSE fillers, lithophilic sites distribution, 3D collector/Li anode structure, and artificial interface layer structure, can effectively balance the relationship between energy density, rate capability, and safety performance. In this review, we first introduce an evaluation system for oriented structures with detailed quantification and definition of each indicator, including tortuosity, homogeneity, anisotropic, and graduality. Then, various preparation techniques for achieving oriented structures in LMBs are categorized, and seven vital factors that need to be considered when choosing among the techniques are elicited. An in‐depth and detailed overview of the mechanisms by which the oriented structure affects the energy density, rate capability, and safety performance of LMBs is presented and can be summarized as follows:

1. For the cathode, enhancing the orientation degree by reducing the tortuosity generally results in more stable electron/ion migration paths that do not vary with the electrode thickness, thus achieving simultaneous improvement in energy density and power density. In addition, the concentration polarization is also significantly reduced due to accelerated mass transport, resulting in a dramatic reduction of anisotropic stress and a more stable structure with the active materials.
2. For SSE, enhancing the degree of orientation by reducing the skeleton/filler's tortuosity and anisotropic generally allows for shortest and continuous ion migration paths to increase the ionic conductivity, which is beneficial for stable cycling at high rates. In addition, the oriented skeleton/filler also improves the interfacial stress/thermal distribution to facilitate the achievement of dendrite‐free Li deposition.
3. For the Li anode and current collector, by designing the structure, introducing oriented interfacial layers, and changing the distribution of lithophilic sites and lithophilic gradient to enhance the orientation degree, a uniform Li+ flow, and controlled deposition pattern can be achieved, which can greatly inhibit the generation of dendrites and improve the safety performance of the LMBs.
4. For separators, uniform Li+ flow and more favorable mechanical properties can be obtained by improving the pore homogeneity or fiber orientation to increase the orientation degree, thus inhibiting dendrite generation and short circuits.

**Figure 20:**
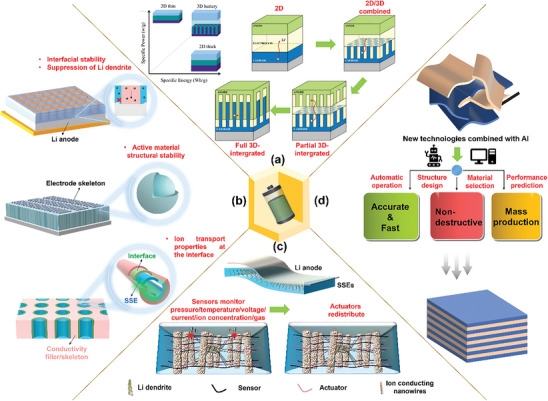
a) Schematic of 2D planar, 2D/3D combined, partially 3D integrated, and fully 3D inter‐embedded solid‐state battery structures. (Reproduced under terms of the CC‐BY license.^[^ ^170^ ^]^ Copyright 2021, Springer Nature) b) Advanced characterization techniques are required to investigate in depth the effect mechanisms of oriented structures. c) Oriented distribution of sensors/actuators to maximize the range and speed of monitor/response for the pressure/temperature/voltage/current/ion concentration/gas changes within LMBs. d) Developing more advanced fabrication techniques of oriented structures in LMBs by incorporating AI.

Despite the great progress made regarding the use of oriented structures in various components of batteries, there are still several challenges associated with their practical application in LMBs. Some of these challenges and future perspectives for the application of LMBs based on oriented modifications include:

1) To synergize optimization strategies for several LMBs’ components, and assembly issues between different 3D‐oriented battery components. As shown in **Figure** **20**a, this self‐assembled, fully 3D battery is subject to very subtle structural adjustments between cathodes, SSE, and anode compared to a 2D planarly assembled battery.^[^ ^170^ ^]^ One of the major advantages of such fully 3D‐oriented integrated batteries is the increase in active substance loading along with the enhanced internal contact surface area between electrodes/electrolytes and the reduction of structural tortuosity, which improves the current output. However, this arrangement exhibits a high degree of complexity, which is expected to be mastered by carefully depositing three specific layers sequentially on top of each other.
2) State‐of‐the‐art characterization techniques are required to investigate in depth the effect of the orientation structure. Although oriented structures can significantly optimize the macroscopic performance of LMBs, the fundamental mechanisms are in many cases not fully elucidated. Therefore, further in‐depth analysis is still required to verify how the interplay between interfacial stability, dendrite suppression, structural stability of active materials, and ion transport properties at the interface is affected by the orientation degree of the oriented structures (Figure 20b).
3) Potential future applications of oriented structures in LMBs are summarized in **Table** **3**:

**Table 3:** Potential future applications of oriented structures in LMBs.

Potential future application	Examples
Flexible batteries/wearable devices	Oriented structures in LMBs enhance mechanical flexibility and resistance to stretching and compression for wearable devices. Flexible electrodes with these structures adapt to battery deformation and twisting.
Recyclable/sustainable batteries	Materials with naturally oriented structures in nature are processed and used in LMBs to reduce the environmental hazards of batteries and improve recyclability.
Battery condition real‐time monitoring system	Precise and oriented sensor alignment: 1) minimizes sensor quantity, reducing cost and complexity; 2) enables real‐time accurate detection and local fast response; 3) facilitates compact battery design and improves mass/volume energy density
Battery self‐conditioning system	Arranging actuators in key positions throughout the battery ensures timely regulation and recovery of its state, including local temperature, pressure, current, and voltage, maintaining stable high performance and preventing safety accidents.
Environmental adaptation	Designing the battery shell and packaging with oriented structures improves environmental adaptability and durability, enhancing anti‐vibration, waterproof, and dustproof properties for diverse environments.
Intelligent charge/discharge control	Combining oriented‐structure sensors with intelligent algorithms enables smart control and optimization of battery charging and discharging. Real‐time state monitoring adjusts parameters to enhance efficiency, extend battery life, and ensure safety and stability.

There is a strong potential to integrate oriented arrangements of sensors/actuators into the LMBs. Sensors based on materials that are piezoelectric/thermoelectric monitor real‐time changes in pressure/temperature/voltage/current/ion concentration/gas of the battery,^[^ ^171^, ^172^ ^]^ while actuators can instantly react to drive the system back from any critical states that arise due to such changes to a safe high‐performance state. Incorporating such smart systems with integrated sensors and actuators can achieve fast cold starts and longer safe cycles of the LMBs.^[^ ^173^ ^]^ Therefore, preparing chemically/electrochemically stable sensors/actuators and orienting them inside the LMBs can maximize the range and response speed with the least quantity of sensors/actuators (Figure 20c).

4) Exploration of advanced techniques to guide an efficient fabrication of internally oriented structures in LMBs. As shown in Figure 17 (see Section 3.8), there is no single ideal method that simultaneously achieves high processing accuracy, fabrication speed, low cost, and nondestructive properties (reducing damage caused by lasers, cutting, etc.) to the battery material. Therefore, it is expected that incorporating AI techniques (Figure 20d) may be effective in solving this complex optimization problem. AI techniques may assist in the preparation of oriented structures in LMBs through data analysis, automatic operation, structure design, material selection, and performance prediction.

## Conflict of Interest

The authors declare no conflict of interest.
